# Systematic Characterization of Antioxidant Shielding Capacity Against Oxidative Stress of Aerial Part Extracts of *Anacardium occidentale*

**DOI:** 10.3390/antiox14080935

**Published:** 2025-07-30

**Authors:** Alejandro Ponce-Mora, Lucia Gimeno-Mallench, José Luis Lavandera, Ryland T. Giebelhaus, Alicia Domenech-Bendaña, Antonella Locascio, Irene Gutierrez-Rojas, Salvatore Sauro, Paulina de la Mata, Seo Lin Nam, Vanessa Méril-Mamert, Muriel Sylvestre, James J. Harynuk, Gerardo Cebrián-Torrejón, Eloy Bejarano

**Affiliations:** 1 Department of Biological Sciences, School of Health Sciences, Universidad Cardenal Herrera-CEU, CEU Universities, 46115 Alfara del Patriarca, Spain; alejandro.poncemora1@uchceu.es (A.P.-M.); lucia.gimenomallench@uchceu.es (L.G.-M.); alicia.domenechbendana@uchceu.es (A.D.-B.); antonella.locascio@uchceu.es (A.L.);; 2 Instituto de Medicina Molecular Aplicada (IMMA), Facultad de Medicina, Universidad San Pablo-CEU, CEU Universities, Campus de Montepríncipe, E-28668 Madrid, Spain; joseluis.lavandera@ceu.es (J.L.L.); irene.gutierrezrojas@ceu.es (I.G.-R.); 3 Department of Pharmacology, Physiology and Neuroscience, Medical School, Rutgers, The State University of New Jersey, 185 South Orange Avenue, Newark, NJ 07103, USA; 4 The Metabolomics Innovation Centre (TMIC), Edmonton, AB T6G 2N4, Canada; rgiebelhaus@uvic.ca (R.T.G.); delamata@ualberta.ca (P.d.l.M.); seolin@ualberta.ca (S.L.N.); james.harynuk@ualberta.ca (J.J.H.); 5 Department of Chemistry, University of Alberta, Edmonton, AB T6G 2N4, Canada; 6 Laboratoire COVACHIM-M2E EA 3592, Université des Antilles, 97157 Pointe-à-Pitre, Francemuriel.sylvestre@univ-antilles.fr (M.S.)

**Keywords:** phytocompounds, *Anacardium*, oxidative stress, antioxidants, NRF2

## Abstract

Oxidative stress is a biological imbalance that contributes to cellular damage and is a major driver of aging and age-related disorders, prompting the search for natural antioxidant agents. Our study is a phytochemical, electrochemical, and biological characterization of the antioxidant potential of aqueous extracts from aerial parts of *A. occidentale*—leaves, bark, fruit, and cashew nuts—traditionally used in folklore medicine. Extracts were analyzed using FT-IR spectroscopy, GC × GC-TOFMS, polyphenol quantification, and antioxidant capacity assays (ABTS, FRAP, DPPH). Biological activity was tested in different mice and human cell lines (SH-SY5Y, MEF, ARPE-19, and HLECs). Aqueous extracts from the leaves and bark of *A. occidentale* exhibited significantly higher antioxidant activity compared to those from the fruit and cashew nut. These extracts showed elevated polyphenol content and strong performance in antioxidant capacity assays. In vitro, leaf and bark extracts enhanced cell viability under H_2_O_2_-induced oxidative stress, preserved mitochondrial membrane potential, and upregulated cytoprotective genes (*HMOX1*, *NQO1*, *GCLC*, and *GCLM*) in multiple cell lines. In contrast, fruit and nut extracts showed minimal antioxidant activity and no significant gene modulation. Our findings underscore the therapeutic potential of *A. occidentale* leaf and bark extracts as effective natural antioxidants and support their further development as candidates for phytotherapeutic interventions.

## 1. Introduction

Due to increasing life expectancy, the global population is aging rapidly, posing significant challenges to public health systems. According to the World Health Organization, by 2030, approximately 1.6 billion individuals will be aged 60 years or older [1]. Advanced age is associated with a heightened risk of developing neurodegenerative disorders, including Alzheimer’s disease, Parkinson’s disease, frontotemporal dementia, amyotrophic lateral sclerosis, and Lewy body dementia, as well as age-related ocular conditions such as macular degeneration, cataracts, and glaucoma [2,3,4,5,6].

Aging is associated with the progressive degeneration of both central and peripheral nervous system tissues, primarily driven by the accumulation of toxic cellular and molecular events. These pathological processes adversely affect brain structure and function, ultimately contributing to a gradual decline in cognitive performance and mental health. Among these molecular events, oxidative stress is at the root of many pathological cellular pathways, playing a pivotal role in human pathology, being critical in the onset and progression of neurodegenerative diseases. High levels of oxidative stress are particularly relevant during aging, as it is believed that a progressive decline in the endogenous antioxidant defenses occurs with age, leading to the accumulation of reactive oxygen species (ROS) and reactive nitrogen species (RNS) [2,3,4]. This is in alignment with the association of oxidative stress with the etiology and progression of multiple age-related diseases [5,6]. Nowadays, the need to identify therapeutic strategies to mitigate the effects of oxidative stress in aging and age-related disorders represents one of the major challenges the scientific community faces.

Oxidative stress refers to the imbalance between the production of harmful reactive species and the ability of an organism to detoxify them through antioxidant cellular capacities. The production of free radicals in an organism is a naturally occurring phenomenon due to cellular metabolism and external factors such as xenobiotics, pollutants, UV radiation, and heavy metals can stimulate the production of harmful oxidative molecules [7]. Optimal redox status is essential for the maintenance of proper cellular function and, when the production of ROS and RNS overwhelms the antioxidant defense, gradual damage is accumulated in cells and tissues. A better understanding of the modulation of the redox status imbalance is imperative for developing antioxidant-based therapies aimed at mitigating disease progression and improving patient outcomes.

Our body possesses both enzyme-based and non-enzymatic mechanisms that contribute to its total antioxidant capacity, which counteract the toxic effects of free radicals. In the antioxidant defense, the nuclear factor erythroid 2-related factor 2 (NRF2) is the best-established master regulator of oxidative stress response—a transcriptional factor modulating the expression level of genes encoding antioxidant and detoxifying enzymes to neutralize the oxidative stress-derived cellular damage [8,9,10]. Some of these cytoprotective genes are involved in glutathione metabolism, such as glutamate-cysteine ligase catalytic subunit (*GCLC*) and glutamate-cysteine ligase modifier subunit (*GCLM*), in heme metabolism, such as heme oxygenase 1 (*HMOX1*), or in detoxifying routes such as NAD(P)H: quinone oxidoreductase-1 (*NQO1*). Of note, a loss of NRF2 activity is considered a pivotal driving force behind the imbalance of redox status and age-related proteotoxicity associated with multiple ageing-related diseases [11].

In recent years, there has been growing interest in identifying compounds derived from plants—also known as phytocompounds—that can exert antioxidant properties, acting as either ROS and RNS scavengers or boosting the transcriptional upregulation of NRF2-target genes. These phytocompounds are often secondary metabolites: non-essential molecules produced by plants that confer the ability to respond to stimuli, adapt to stress, and provide protection against oxidative stress. Glycosides, alkaloids, terpenoids, and polyphenols are some of these metabolites, and the latter are now being deeply investigated for their promising pharmacological properties [12,13,14].

In this regard, ethnobotany becomes especially important for gathering all information related to the properties, therapeutic potential, and use of plants by societies and communities. Medicinal plants constitute one of the most widely utilized forms of traditional medicine. For many communities, herbal remedies often serve as a primary—or even sole—means of accessing healthcare. The therapeutic properties of many plants have not been thoroughly researched and analyzed from a scientific perspective. The combination of ethnobotanical knowledge and omics technologies has already been proposed as a promising method to identify new phytocompounds or evaluate the potential of different plant extracts [15].

There is considerable interest in identifying bioactive compounds capable of counteracting pathophysiological conditions associated with oxidative stress. A key preclinical step in this process involves evaluating the antioxidant potential of phytochemicals or plant extracts using cellular models derived from tissues particularly vulnerable to oxidative damage, such as the retina, lens, or brain. In this context, one of the most widely used in vitro models is the SH-SY5Y cell line, derived from human neuroblastoma, which has been extensively employed in neurodegeneration research. This cell line is considered a reliable and well-established model for investigating the protective effects of bioactive compounds against oxidative stress-induced cytotoxicity, due to its neuronal characteristics and sensitivity to oxidative insults [16].

Growing evidence suggests that the family *Anacardiaceae*, which comprises approximately 81 genera and around 800 species, might be a promising source of bioactive phytochemicals and nutraceuticals [17]. In most cases, these are tropical plant species, typically exhibiting arboreal or shrubby growth forms, and different members of the family have garnered the scientific community’s attention, such as *Pistacia vera*, *Mangifera indica*, or *Anacardium occidentale*. The latter is a tree about 5 to 7 m tall, native to South America, specifically the northwest of Brazil. However, due to the agri-food interest in the cashew nut, *A. occidentale* is now cultivated in other tropical regions such as Africa, Asia, and India. Apart from the cashew nut, a product well-known worldwide as a food snack, the *A. occidentale* fruit, known as the cajú apple, is often consumed as juice, jam, or simply as a fresh fruit when mature. Additionally, leaves and bark are used in folklore medicine to treat different ailments, and some studies suggest that these two aerial parts seem to possess certain antidiabetic and antihyperglycemic activities [18,19].

Despite the pharmaceutical potential of *A. occidentale* being explored, there is fragmented knowledge about the antioxidant potential of the different aerial parts. This work develops a multidisciplinary approach to analyze the antioxidant properties of the aqueous extract of four aerial parts of the plant (leaves, bark, fruit, and cashew nuts) and their protective effects against H_2_O_2_-derived toxicity. To assess their therapeutic potential, the cytotoxicity and neuroprotective effects of the extracts were evaluated in SH-SY5Y neuroblastoma cells subjected to H_2_O_2_-induced oxidative stress. Additionally, the expression levels of endogenous defense-related genes were analyzed to further elucidate the underlying protective mechanisms.

## 2. Materials and Methods

### 2.1. Preparation of Plant Extracts

The aerial parts of *A. occidentale* (leaves, bark, fruit, and cashew nut) were harvested from a local crop field (16°07′29.1″ N 61°34′53.9″ W) in Goyave (Guadeloupe (FWI)). Then, 374 g of dried leaves was introduced into a microwave extractor (ETHOS X advanced extraction system (Milestone**^®^**, Bergamo, Italy)) chamber containing 1.5 L of distilled water. After the extraction, the residue was lyophilized using a freeze-dryer, model Alpha 1–4 LD plus (Martin Christ**^®^**^,^ Germany). After lyophilization, 11.98 g of dried leaf extract was obtained, corresponding to a 3.2% yield. For the bark extract, 1009.2 g of *A. occidentale* bark was introduced into a microwave extractor chamber containing 1.5 L of distilled water. After the extraction, the residue was lyophilized. After the lyophilization, 41.29 g of dried bark extract were obtained, corresponding to a 4.09% yield. The starting material for the obtention of the aqueous *A. occidentale* extract was 125.21 g of lyophilized fruits. The material was smashed and introduced into a microwave extractor chamber containing 1.5 L of distilled water. Analogous to the bark aqueous extract, a microwave-assisted extraction was performed. The remaining material underwent lyophilization. After the lyophilization process, 61.33 g of dried fruit extract was obtained, corresponding to a 48.98% yield. Then, 1 kg of dried *A. occidentale* nuts was introduced into a microwave extractor chamber containing 1.5 L of distilled water. A microwave-assisted aqueous extraction was performed. After the extraction, the residue was lyophilized. After the lyophilization, 94.86 g of dried nuts extract was obtained, corresponding to a 9.5% yield.

### 2.2. Metabolomic Profile

#### 2.2.1. Fourier Transform Infrared Spectroscopy (FT-IR)

Fourier Transform Infrared Spectroscopy (FT-IR) was used for the preliminary phytochemical characterization of plant extracts. This technique identifies functional groups and molecular structures based on vibrational transitions, providing insights into the chemical composition of complex mixtures. Samples for FT-IR analysis were prepared by dissolving four distinct plant extracts in distilled water to achieve a uniform concentration of 1 mg/mL, ensuring optimal signal intensity while minimizing saturation effects. Spectral data acquisition was performed with the Spectrum Two FT-IR spectrometer (Perkin Elmer, Madrid, Spain), equipped with an Attenuated Total Reflection (ATR) accessory for analyzing liquid and semi-solid samples, allowing for direct measurement without extensive preparation. Each extract was analyzed separately, and spectra were recorded over the mid-infrared range (4000–400 cm^−1^). After acquisition, raw spectra underwent baseline correction and normalization using Spectrum 10™ software (Perkin Elmer), which is essential for minimizing noise and enhancing peak identification accuracy. Characteristic water absorption bands were computationally removed to prevent interference with phytochemical signal interpretation. Key absorption bands were identified and assigned to specific functional groups like hydroxyl (-OH), carbonyl (C=O), alkene (C=C), and amine (N-H) groups, indicative of phytochemical classes such as flavonoids, alkaloids, terpenoids, and phenolic compounds.

#### 2.2.2. Determination of the Total Polyphenol Content (TPC)

The total polyphenol content (TPC) of the four extracts was determined by the Folin–Ciocalteu method. Briefly, 1 mg/mL solutions of each extract were prepared by dissolving the dried extract in distilled water. The solutions were filtered to avoid the presence of undesired particles. The reaction was carried out by mixing 100 µL of each 1 mg/mL aqueous extract, 600 µL of distilled water, 600 µL of sodium carbonate 7.5%, and 200 µL of the Folin–Ciocalteu reagent. The mixture was incubated for 10 min at 50 °C. The absorbance was measured at a wavelength of 760 nm using the spectrophotometer Genesys 20 (Thermo Scientific, Waltham, MA, USA). For the analysis, a standard curve using gallic acid 2.5 mM was used, and the results were expressed as gallic acid equivalents (GAEs). Three experiments were conducted in triplicate to ensure the reliability and reproducibility of the results.

#### 2.2.3. GC × GC-TOFMS Analysis

##### Derivatization Analysis

Samples were prepared and analyzed as previously described [20] with minor modifications. Analytes were extracted in 1 mL 50:50 methanol (Optima Grade, Fisher Scientific, Hampton, NH, USA) and chloroform (HPLC-grade, Fisher Scientific) in a 2 mL microcentrifuge tube (VWR, Radnor, PA, USA). The extraction was vortexed for 5 min, then centrifuged at 10,000 rpm for 10 min (MIKRO 185, Hettich Zentrifugen, Westphalia, Germany). The supernatant was then removed and diluted by a factor of 40; then, 750 μL of the diluent was aliquoted into a 2 mL clear-glass GC vial (Chromatographic Specialties) and capped. The extract was then dried under nitrogen at 40 °C. A 100 μL aliquot of toluene dried with Na_2_SO_4_ was added to the sample and dried under nitrogen at 50 °C. A 50 μL aliquot of 20 mg/mL of CH_3_ONH_2_ · HCl (Fisher Scientific) in pyridine (HPLC Grade, Fisher Scientific) was added to the vial and incubated in the heating block for 1 h at 60 °C. Following this, a 75 μL aliquot of MSTFA + 1% TMCS (Fisher Scientific) was added to the vial, and the sample was again incubated for 1 h at 40 °C. The sample was then transferred into a 300 μL glass insert autosampler vial (Chromatographic Specialties) and capped.

##### Untargeted GC × GC-TOFMS

Untargeted GC × GC-TOFMS metabolomics was performed using a method reported previously [21]. A total volume of 1 µL of each sample was injected using a MultiPurpose Sampler (MPS; Gerstel, Linthicum Heights, MD, USA) into a CIS-4 inlet (Gerstel) operated in splitless inlet mode at 250 °C. Separation and analysis were performed with a LECO BT 4D GC × GC-TOFMS (LECO, St. Joseph, MI, USA). The first-dimension chromatographic column was a 60 m × 0.25 mm × 0.25 μm Rxi-5SilMS (Chromatographic Specialities, Brockville, ON, Canada), and the second-dimension column was a 1.4 m × 0.25 mm × 0.25 μm Rtx-200MS (Chromatographic Specialities). Ultra-pure helium gas (Linde Canada Inc., Edmonton, AB, Canada) was used as the carrier gas at a constant flow rate of 2.0 mL/min. The initial oven temperature was held at 80 °C for 4 min before ramping up to a final temperature of 315 °C at a rate of 3.5 °C/min, where it was held for 10 min. The secondary oven and modulator temperature offsets were set at +10 °C relative to the GC oven and +15 °C relative to the secondary oven, respectively. The modulation period was 2.5 s. Data acquisition was conducted at a rate of 200 Hz over a mass range between 40 and 800 *m*/*z*. A detector voltage was applied, and an electron impact energy of −70 eV was set for ionization. The mass spectrometer transfer line and ion source temperatures were set to 250 °C and 200 °C, respectively. The total chromatographic analysis time was 81.1 min for each run. A reagent blank and instrument blanks were performed for quality control purposes.

##### Data Processing

The GC × GC-TOFMS data were processed using LECO ChromaTOF**^®^** for BT (v5.58.05; Leco). Peak finding was performed with an S/N above 1000 and a minimum stick count of three. Subpeaks were combined with a mass spectral match factor over 500. Peaks eluting within 0.8 s to 1.1 s in the second dimension were removed, as they elute in the column bleed. Retention indices for all detected peaks were calculated based on the retention time of linear alkanes (C7–C30) analyzed on the same day. Library matching for putative compound identification was performed against commercially available and in-house databases. A minimum mass spectral similarity of 800 and a retention index within ±30 units was required to assign a putative identity. The compounds trans-catechin, gallic acid, protocatechoic acid, and pyrogallol were putatively identified by using a Target Analyte Search in ChromaTOF**^®^** with a S/N of 750, a minimum area of 100, and a minimum height of 25. All analytes were putatively identified according to level 2 of the metabolomics standards initiative [22]. Analytes of interest were normalized to the total peak area of all detected peaks in those samples [23].

### 2.3. Electrochemical Analysis

#### 2.3.1. Cyclic Voltammogram

Electrochemical experiments were conducted as previously published [24,25]. The solutions of different extracts were previously prepared by dissolving 100 mg of the target extract in 10 mL of 10 mM CuSO_4_ solution. Cyclic voltammograms were recorded at room temperature in CH I660 equipment (CH Instruments, Austin, TX, USA) working with a conventional three-electrode cell using a platinum wire auxiliary electrode, an Ag/AgCl reference electrode, and a glassy carbon electrode (GCE) as a working electrode. The study of the cyclic voltammogram signals corresponding to the cathodic region, attributed to the reduction of Cu^2+^ to Cu^0^, is observed at −0.65 V versus Ag/AgCl. As a result of the reduction, a deposit of Cu^0^ takes place in the GCE (stripping). In the subsequent anodic scan, the deposit of solid Cu^0^ is oxidized to Cu^2+^, producing the stripping signal corresponding to the oxidation peak at +0.20 V. The study of the modification of both signals indicates the chelation of copper exerted by the different extracts.

#### 2.3.2. Electrochemical Antioxidant Activity Determination

The total antioxidant capacity was measured using the BRS device following the manufacturer’s instructions (BQC Redox Technologies, Oviedo, Asturias, Spain). This portable instrument enables the assessment of the total antioxidant capacity (TAC). Disposable strips interacting with the sample generate an electrochemical signal proportional to the redox activity [26]. Measurements were expressed as BRS values in microcoulombs (µC), and experiments were performed in triplicate.

### 2.4. Antioxidant Analysis

#### 2.4.1. ABTS Assay

As an initial approach, the assay was performed at a concentration of 1 mg/mL for each aqueous extract using the ABTS Assay Kit (Bioquochem, Asturias, Spain) according to the manufacturer’s instructions using the spectrophotometer Genesys 20 (Thermo Scientific). Trolox was used as a standard, and data were shown as Trolox Equivalents Antioxidant Capacity (TEAC) (µM Trolox). The ABTS+ radical scavenging capacity was calculated as follows:

$$\%\;\mathrm{Inhibition}=\left(1-\frac{A_{s}}{A_{o}}\right)\times 100$$

where $A_{o}$ is the blank-corrected absorbance of standard 1 (0 µM Trolox) and $A_{s}$ is the blank-corrected absorbance measured for each sample.

For a more in-depth study, the 2,2′-azino-bis(3-ethylbenzothiazoline-6-sulfonic acid) (ABTS) assay was performed on each *A. occidentale* aqueous extract as previously published [27]. First, different concentrations of each aqueous extract were tested to assess the IC_50_ value. The ABTS+ radical was produced by reacting 2,2′-azino-bis(3-ethylbenzthiazoline-6-sulphonic acid (ABTS) 7 mM with K_2_S_2_O_8_ 2.45 mM (both reagents dissolved in water) at a volume ratio of 1:1. After mixing, the reaction was incubated in the dark at room temperature for 16 h. The radical was diluted in ethanol to reach a final absorbance of 0.750 ± 0.025 at 734 nm. For this experiment, different concentrations of the aqueous extracts were tested: 0.0001, 0.001, 0.01, 0.1, 1, 10, 100, 1000, and 10,000 µg/mL in a 96-well plate. Then, 150 µL of each solution was added to the plate, followed by 50 µL of the ABTS^+^ radical. The plate was then incubated for 30 min at room temperature. After incubation, the absorbance was measured at a wavelength of 734 nm using a Varioskan LUX plate reader (Thermo Fisher Scientific, Vantaa, Finland).

#### 2.4.2. FRAP Assay

An initial assay was performed at a concentration of 1 mg/mL for each aqueous extract, using the FRAP Assay Kit (Bioquochem, Asturias, Spain), according to the manufacturer’s instructions using the spectrophotometer Genesys 20 (Thermo Scientific, Waltham, MA, USA). A more detailed study was performed, in which the Ferric Reducing Antioxidant Power (FRAP) assay of four different extracts of aerial parts of *A. occidentale* was evaluated, as previously described in the literature [27]. The FRAP reagent was prepared by mixing 10 mM 2,4,6-tripyridyl-S-triazine in 40 mM HCl, 20 mM FeCl_3_·6H_2_O and 300 mM acetate buffer, all dissolved in Milli-Q water, in a proportion of 1:10:10. Briefly, 180 µL of the FRAP reagent was added to each well of a 96-well plate, followed by the addition of 20 µL of each aqueous extract solution. Concentrations of 0.0001, 0.001, 0.01, 0.1, 1, 10, 100, 1000, and 10,000 µg/mL of the aqueous extract were tested. After a 30 min incubation period in the dark, the absorbance at 593 nm was measured using a Varioskan LUX plate reader (Thermo Fisher Scientific, Vantaa, Finland).

#### 2.4.3. DPPH Assay

The assay was initially performed at a concentration of 1 mg/mL for each aqueous extract using the DPPH Assay Kit (Bioquochem, Asturias, Spain) according to the manufacturer’s instructions using the spectrophotometer Genesys 20 (Thermo Scientific, Waltham, MA, USA). Trolox was used as a standard, and data are shown as Trolox Equivalents Antioxidant Capacity (TEAC) (µM Trolox). The DPPH radical scavenging capacity was calculated as follows:

$$\%\;\mathrm{Inhibition}=\left(1-\frac{A_{s}}{A_{o}}\right)\times 100$$

where $A_{o}$ is the blank-corrected absorbance of standard 1 (0 µM Trolox) and $A_{s}$ is the blank-corrected absorbance measured for each sample.

A more detailed study was conducted in which a 2, 2-diphenyl-1-picrylhydrazyl (DPPH) assay was performed for each *A. occidentale* aqueous extract as published [25]. The DPPH radical was generated by preparing a DPPH 0.2 mM solution in absolute ethanol. Different concentrations of the aqueous extract were tested: (0.0001, 0.001, 0.01, 0.1, 1, 10, 100, 1000, 10,000) µg/mL in a 96-well plate. Then, 150 µL of each aqueous extract solution was added to the plate, followed by the addition of 50 µL of the DPPH radical. The plate was then incubated for 30 min at room temperature. Absorbance was measured at a wavelength of 517 nm using the Varioskan LUX plate reader (Thermo Fisher Scientific, Vantaa, Finland).

### 2.5. Cell Culture

Human lens epithelial cells (HLECs, line SRA 01/04) were kindly donated by Dr. Venkat Reddy, (University of Michigan, Ann Arbor, MI, USA). Mouse embryonic fibroblasts (MEFs) from mice WT were generously donated by Dr. Masaaki Komatsu (Juntendo University, Japan). Human retinal pigment epithelial cells (ARPE19) and SH-SY5Y human neuroblastoma cells were purchased from the American Type Culture Collection (ATCC). These three cell lines were cultured in Dulbecco’s Modified Eagle Medium (DMEM) (GIBCO) containing 10% fetal bovine serum (FBS), 1% penicillin-streptomycin mix (10,000 U/mL), 1% MEM non-essential amino acids (GIBCO), and 1% sodium pyruvate 100 mM (GIBCO) in an incubator at 37 °C in a humid atmosphere containing 5% CO_2_.

### 2.6. Analysis of Cytotoxicity and Cell Viability Against Oxidative Stress

#### 2.6.1. Cytotoxicity Activity

A density of 5 × 10^3^ cells per well was used to seed the SH-SY5Y cells in a 96-well plate, which was then grown in the incubator for 24 h. The cells were exposed to different concentrations (ranging from 0.01 to 1000 µg/mL) of an aqueous leaf and bark extract, which had been resuspended in 50 mM PBS for 24 h. Following this pretreatment with the plant extracts, the 3-(4,5-dimethylthiazol-2-yl)-2,5-diphenyl tetrazolium bromide (MTT) assay was conducted to assess cell proliferation and viability. Then, 5 μL of 20 mg/mL of MTT was added to the wells, and the plate was incubated at 37 °C for 2 h; after this, 200 μL of dimethylsulfoxide was added to solubilize the formazan crystals, and the plate was left in the dark at room temperature for 30 min. Absorbance was measured at 570 nm using a Varioskan LUX plate reader (Thermo Fisher Scientific, Vantaa, Finland). Dose–response curves were plotted using linear regression analysis and LD50 (inhibition of cell viability) concentrations were calculated.

#### 2.6.2. Neuroprotection Assay Against H_2_O_2_-Induced Cytotoxicity

As in the previous experiment, 5 × 10^3^ SH-SY5Y cells/well were seeded in 96-well plates and grown in the incubator for 24 h. Cells were pretreated with cell media or 5, 10, 20, 50, 100, and 200 µg/mL of the PBS 1×-resuspended aqueous bark and leaf extract. After 24 h, except for control wells, cells were exposed for four hours to H_2_O_2_ at a concentration of 500 µM. The MTT viability assay was performed according to the conditions described above. Cell viability values were expressed as a percentage in comparison to the viability of the untreated cells.

#### 2.6.3. Determination of the Levels of Intracellular ROS and RNS

Dichlorofluorescein diacetate (DCFH2-DA) was used to determine intracellular ROS and RNS levels as previously reported [27]. Intracellular esterases cleave DCFH2-DA to form the fluorescent compound DCF. The fluorescent intensity is proportional to the intracellular ROS and RNS level. Then, 5 × 10^5^ SH-SY5Y cells were seeded in 96-well culture black, clear-bottom plates and, after 24 h, were treated with the test extract for additional 24 h. Then, the cells were loaded with 10 µM DCFH_2_DA in FBS-free culture medium for 50 min in the dark at 37 °C in a CO_2_ incubator. Finally, the cells were exposed to 1 mM H_2_O_2_ for four hours. After the incubation period, the cells were twice washed with Hank’s buffer. Fluorescence was measured using a Varioskan LUX microplate reader (Thermo Fisher Scientific, Vantaa, Finland) with excitation at 485 nm and emission at 535 nm wavelengths. Results are expressed as a percentage of the fluorescent increase compared to the control. All experiments were performed at least four times in sextuplicate for each extract concentration.

#### 2.6.4. Determination of the Mitochondrial Membrane Potential

Furthermore, 96-well plates were seeded with 5 × 10^3^ SH-SY5Y cells per well and incubated for 24 h. The cells were pretreated with different concentrations (5, 10, 25, 50, 100 and 200 µg/mL) of the aqueous leaf and bark extract for 24 h. Following the treatment with plant extracts, cells were treated with 500 µM H_2_O_2_ for 4 h, after which they were washed twice and incubated for 20 min with 5 µM JC-1 in culture media in the dark. The plates were then washed three times with Hank’s buffer and left in 200 µL of fresh buffer. The absorbance of each well was measured at excitation wavelengths of 535 nm and emission wavelengths of 595 nm, as well as at emission wavelengths of 485 nm and 535 nm, to evaluate the presence of JC-1 aggregates and assess red and green color, respectively.

Flow cytometry studies were also conducted to evaluate the mitochondrial membrane potential. Six-well plates were seeded with 5 × 10^5^ SH-SY5Y cells per well. After 24 h, the cells were treated with 100 or 200 µg/mL of aqueous extract from the leaves or bark and incubated for a further 24 h. Then, cells were treated with 400 µM H_2_O_2_ for 4 h, after which they were washed twice with Hank’s buffer and incubated for 20 min in the dark with 5 µM JC-1 in culture media. After staining, the cells were washed three times with Hank’s buffer, resuspended in 500 µL of culture medium and analyzed using flow cytometry with an Accuri C6 flow cytometer (BD Biosciences, Ann Arbor, MI, USA).

### 2.7. Quantitative Real-Time PCR Assays

100 mm TC-treated culture dishes were seeded with either HLECs, MEFs, or ARPE19 cells. Cells were left growing until they reached a confluence of 80%. Then, the cells were washed with PBS 1X, and fresh media containing a concentration of 1 mg/mL of the extract of leaves, bark, fruit, or cashew nut of *A. occidentale* was added. Cells were incubated at 37 °C for 18 h with each plant extract or mock solution. Then, the cells were washed once with cold PBS 1X and scraped in the same buffer, and the cell pellets obtained by low-speed centrifugation were stored at −80 °C until processing. Total RNA was isolated according to the manufacturer’s instructions. Following this, 1 μg of total RNA of each sample was transcribed using the NZY Reverse Transcriptase kit (NZYTech, Lisboa, Portugal). Quantitative real-time PCR (qRT-PCR) was employed to assess the mRNA expression levels of the following genes: *HMOX1*, *NQO1*, *GCLC* and *GCLM*. The primers used for each gene are shown in Table 1. qPCR experiments were performed employing the QuantStudio™ 5 Real-Time PCR System. The relative expression of the gene *GAPDH* was used as housekeeping to normalize target expression vs. sample condition.

### 2.8. Statistical Analysis

All numerical results are reported as the mean ± SEM from a minimum of three independent experiments. In all instances of “n” refer to individual experiments, indicated in the corresponding figure legends. GraphPad InStat software (GraphPad) was used for analysis of statistical significance. Two-tailed Student’s *t* test was used to evaluate single comparisons between different experimental groups. Differences were considered statistically significant for a value of *p* < 0.05 and are denoted by an asterisk in the graph. Comparisons were made between control and treated groups or the entire intragroup using one-way ANOVA or two-way ANOVA and Dunnett’s multiple comparisons test using GraphPad Prism 10.0 (GraphPad-Software, La Jolla, CA, USA).

## 3. Results

### 3.1. Metabolomics Revealed Differential Polyphenolic Content in the Extracts of the Aerial Parts of A. occidentale

**Figure 1:**
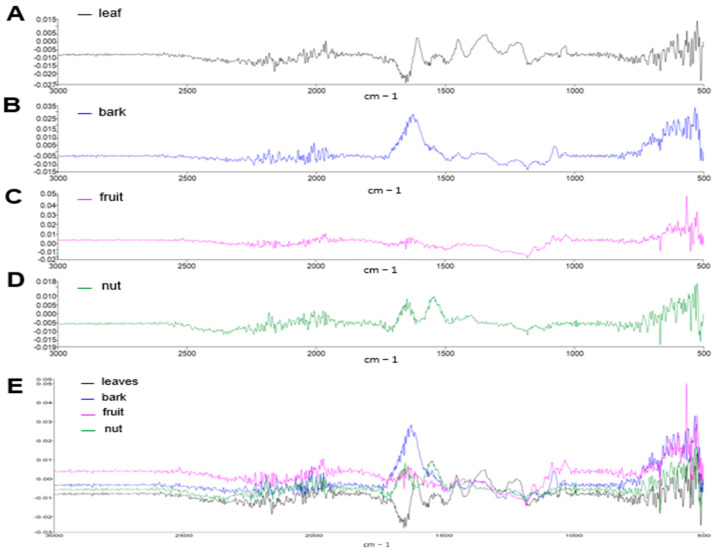
Differential FT-IR spectra profiles in aqueous extracts of the aerial part of *Anacardium occidentale*. FT-IR spectra profiles of different aqueous extracts of *Anacardium occidentale* (**A**) leaf (black), (**B**) bark (blue), (**C**) fruit (pink), and (**D**) nut (green) are shown. (**E**) Composite of FT-IR spectra profiles.

The FT-IR analysis showed meaningful differences between the spectra of the tested materials. The relative intensities differed, and each spectrum provided essential information about specific functional groups (Figure 1). The spectra of the four extracts were integrated to generate a composite FT-IR spectrum, which represents the plant’s phytochemical profile, capturing both shared and unique functional groups (Figure 1A). The leaf extract presented a modest signal in the region of ~1600 cm^−1^, suggesting the presence of aromatic C=C or any carbonyl groups (e.g., phenolic acids). In the case of the bark spectrum, a prominent peak around the region of ~1600 cm^−1^ was detected, which may indicate the presence of carbonyl groups (C=O) or aromatic C=C. Additionally, the spectral complexity in the region 1000–1300 cm^−1^ might be potentially attributable to lignin, a characteristic component of plant bark. The spectrum corresponding to the fruit sample showed a flat signal profile up to 1300 cm^−1^ area and the signal increased around 500–800 cm^−1^. This event can be associated with the presence of sugars, polysaccharides, or organic acids, which are classic components of fruits. The lack of signal in the 1600 cm^−1^ region of the spectrum suggests the absence of conjugated carbon-carbon or phenolic compounds. The spectrum corresponding to the nut extract showed a low profile in the 1600 cm^−1^ region. Still, it contained structures in the 1000–1300 cm^−1^ region, indicating that the sample could be rich in fats, proteins, or simple phenols.

**Figure 2:**
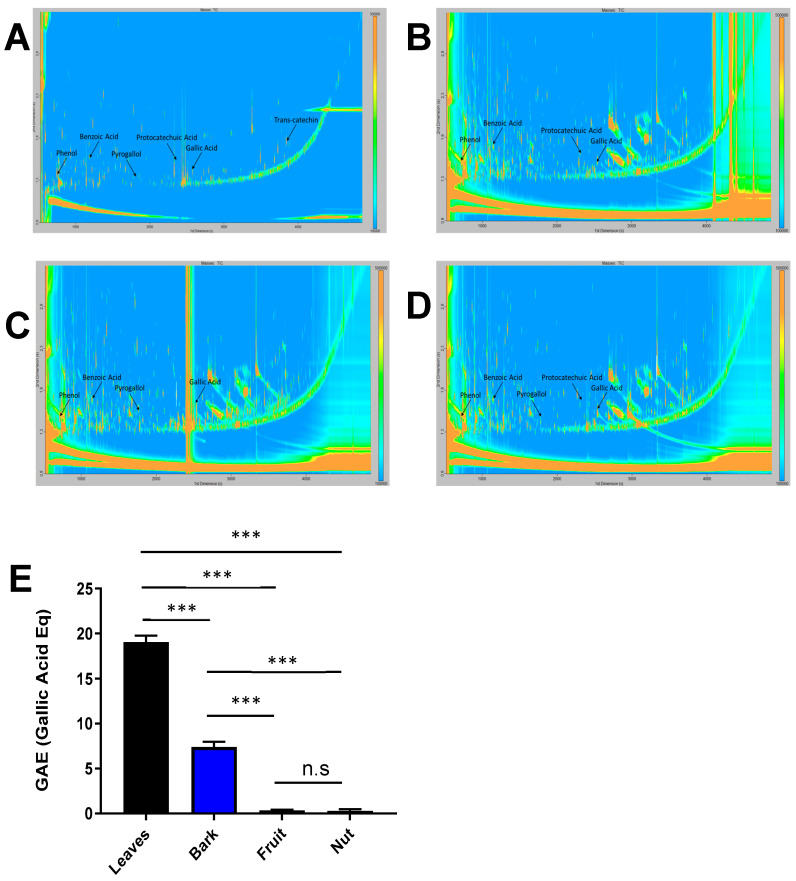
Metabolomics reveals differential polyphenol composition in aqueous extracts of the aerial part of *Anacardium occidentale*. GC × GC-TOFMS profiles for (**A**) leaf, (**B**) bark, (**C**) fruit, and (**D**) nut extracts. Specific polyphenols are indicated in each profile. (**E**) Total phenolic content for leaf (black), bark (blue), fruit (pink), and nut (green) aqueous extract of *Anacardium occidentale* n = 3; ***, *p* < 0.001, *t* test.

Next, we analyzed the presence of polyphenols in the extracts of the aerial parts of *A. occidentale* (Figure 2). The leaf extract was highly enriched in polyphenols with 19.08 ± 0.67 µg of gallic acid equivalent (GAE)/mL, followed by the bark extract, with a content of polyphenols of 7.41 ± 0.55 µg GAE/mL. The values for fruit and cashew nut were low. The content of polyphenols in the fruit and cashew nut extract were 0.35 ± 0.09 µg GAE/mL and 0.30 ± 0.16 µg GAE/mL, respectively. GCxGC-TOFMS revealed the presence of specific polyphenols in the different extracts (Figure 2, Tables S1–S4). Phenol, benzoic acid, and gallic acid were identified in all four samples (Figure S1, Tables S1–S4). In the leaves, aqueous extract trans-catechin, protocatechoic acid, and pyrogallol were all putatively identified. Interestingly, trans-catechin was only found in the leaf extract (Figure 2, Figure S1, Tables S1–S4).

### 3.2. An Array of Antioxidant Capacity Assays Revealed Differential Antioxidant Potential of the Aerial Parts of A. occidentale

**Figure 3:**
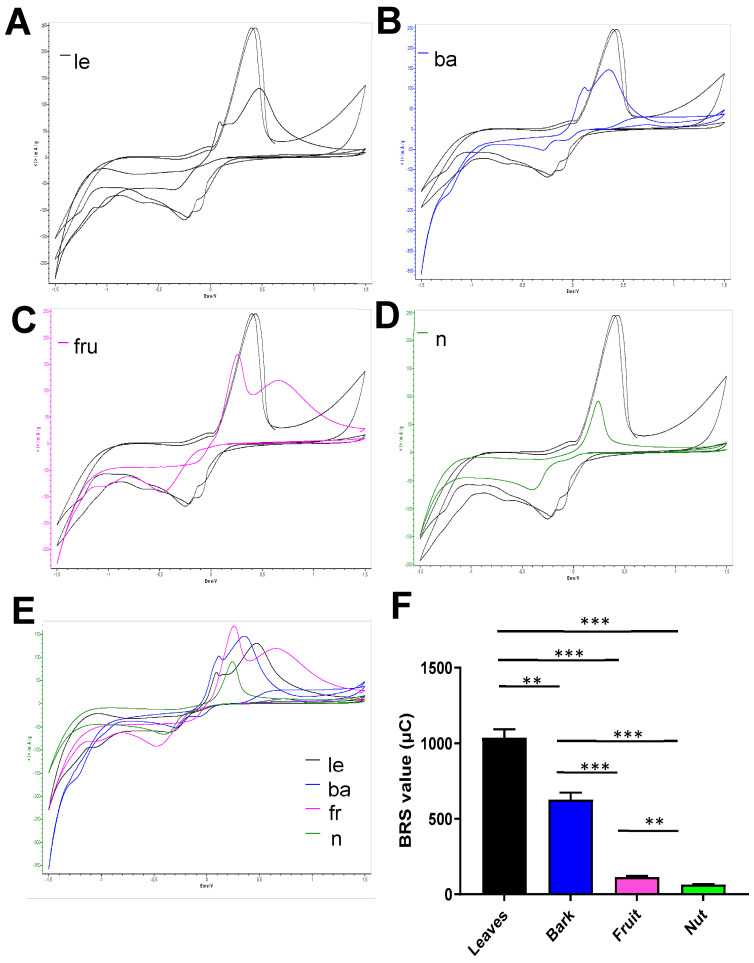
Electrochemical analysis reveals differential antioxidant capacity of the aerial part of *Anacardium occidentale*. Cyclic voltammograms of films originating from leaf (**A**), bark (**B**), fruit (**C**), and nut (**D**) aqueous extract of *Anacardium occidentale*. Overlapping of the different profiles is shown (**E**). Total antioxidant activity was using the BRS device (**F**). Measurements are expressed in microcoulombs (µC). n = 3; **, *p* < 0.01; ***, *p* < 0.001, *t* test.

To gain information about the electroactivity of the extracts, the cyclic voltammetry technique based on copper chelation was carried out [28]. Cyclic voltammetry revealed differential electroactive properties of the different samples (Figure 3A–E). A cathodic shift to a higher reduction potential of the signal of Cu^2+^ reduction compared with the copper control profile was observed in all the samples. This perturbation of the cathodic signal is attributed to the chelation of Cu^2+^ by the metabolites present in the extracts. The modification was lower in the case of nut extract (Figure 3D). When the oxidation of the stripping Cu^0^ was analyzed, extract of leaves, bark, and fruit showed a splitting of the signal, confirming the chelation detected in the cathodic signal (Figure 3A–C). In the case of the nut extract, this anodic signal was very similar to the copper control (Figure 3D), suggesting no evidence of the coordination of this extract with copper. Electrochemical quantification of the total antioxidant capacity revealed significant differences (Figure 3E). The value for the leaf extract was 1039 ± 54.50 µC, and the bark extract was approximately half (627.2 ± 45.85 µC). The values for the fruit and the cashew nut were significantly lower, with 114.2 ± 7.82 µC and 64.02 ± 2.97 µC, respectively, although the fruit extract was significantly different to the nut sample.

**Figure 4:**
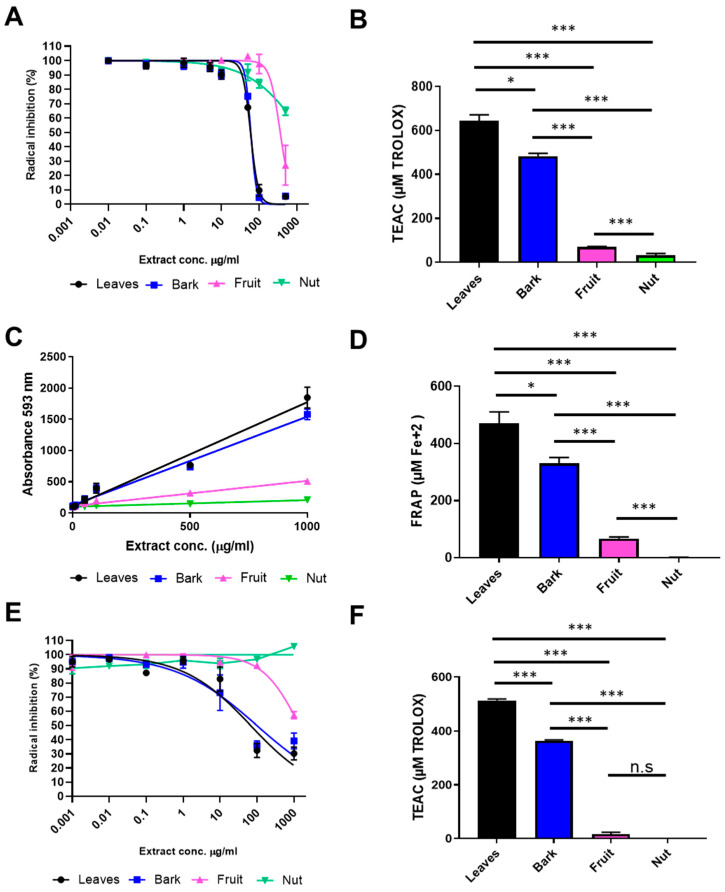
Antioxidant capacity assay of aerial part of *A. occidentale*. (**A**,**B**) ABTS (2,2′-azino-bis(3-ethylbenzothiazoline-6-sulfonic acid)), (**C**,**D**) Ferric Reducing Antioxidant Potential (FRAP), and (**E**,**F**) 2,2-diphenyl-1-picrylhydrazyl (DPPH) assays were performed in leaf (black), bark (blue), fruit (pink), and nut (green) aqueous extracts of *A. occidentale*. TEAC: Trolox Equivalents Antioxidant Capacity n = 3; *, *p* < 0.05; ***, *p* < 0.001, *t* test.

Next, we carried out a set of broadly used assays to measure scavenging and antioxidant capacity: 2,2′-azino-bis-3-ethylbenzthiazoline-6-sulphonic acid (ABTS) (Figure 4A,B) and Ferric Reducing Antioxidant Power (FRAP) assay (Figure 4C,D). Two methods based on electron transfer capacity, while 2,2-diphenyl-1-picryl-hydrazyl-hydrate (DPPH) (Figure 4E,F) is based on hydrogen atom transfer capacity. Inhibition curves were plotted for dose-dependent experiments (Figure S2), and IC_50_ values were determined along with Trolox Equivalent Antioxidant Capacity (TEAC) values—a well-defined measurement of antioxidant strength. Quantitative parameters corresponding to these antioxidant assays are shown in Table 2.

Leaf extract had the higher ABTS^•+^ radical scavenger activity (644.40 ± 27.60 TEAC (µM Trolox)). The bark extract showed slightly lower activity (482.70 ± 13.68 TEAC), while the scavenging activity of the fruit and nut samples were only 69.97 ± 2.12 and 32.34 ± 7.71 TEAC, respectively (Figure 4A). Both leaves and bark extracts of *A. occidentale* exhibited high antioxidant activity, with IC_50_ values of 12.3 ± 2.1 and 13.8 ± 4.1 µg/m, respectively (Figure 4B). On the contrary, low inhibitory activities against the radical ABTS^•+^ were found in fruit and nut extracts. In the case of the fruit extract, the IC_50_ value was 447.2 ± 15.0 µg/mL, while the value for the nut sample was undefined and >1000 µg/mL (Figure 4B, Table 2).

In alignment with the previous results, similar antioxidant behavior was found when the FRAP assay was carried out. The FRAP assay using 1 mg/mL concentration revealed differential antioxidant properties for leaves and bark extracts (Figure 4C). The FRAP value of leaf extract was 470.30 ± 40.44 µM Fe^2+^, and bark extract showed an antioxidant value of 331.10 ± 19.41 µM Fe^2+^. These FRAP values were comparatively higher than those observed for the fruit and nut extracts, which were 67.10 ± 5.69 and 0.62 ± 0.62 µM Fe^2+^, respectively. Leaf and bark extracts were the most antioxidants samples, with values of 0.13 and 0.12 of TEAC, respectively. The antioxidant capacity of fruit and nut extract was close to zero in this assay, with values of 0.03 and 0.01 of TEAC (Figure 4D, Table 2).

The DPPH assay showed a similar pattern on radical scavenging capacity (Figure 4E,F). TEAC values for DPPH using 1 mg/mL concentration were 513.30 ± 5.64 µM Trolox for leaf extract, 364.7 ± 2.52 µM Trolox for bark extract, 16.95 ± 7.03 µM Trolox for fruit extract, and close to 0 µM Trolox for nut extract (Figure 4E). In this case, the IC_50_ values of the leaves (58.67 µg/mL) and bark (79.52 µg/mL) extracts were significantly higher when compared to fruit and nut samples, the latter with IC values higher than 1000 µg/mL (Figure 4F, Table 2).

In sum, a set of different assays that quantify the H-donating activity and electron transfer ability revealed that leaf and bark aqueous extracts of *A. occidentale* had significantly higher antioxidant potential than fruit and nut aqueous extracts.

### 3.3. Leaf and Bark Extracts of A. occidentale Are Protective Against Oxidative Stress in SH-SY5Y Cells

**Figure 5:**
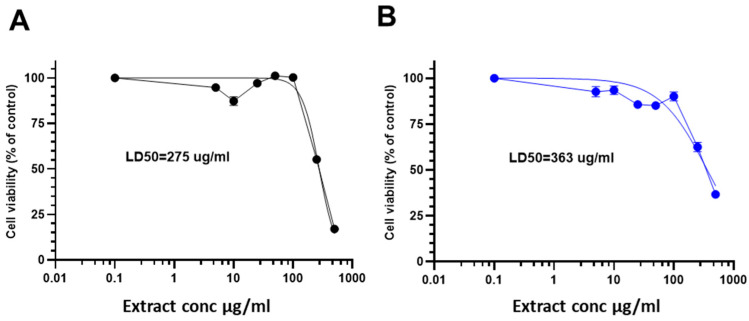
Cytotoxicity of leaves and bark extracts in SH-SY5Y cells. Cell viability was analyzed using MTT assay. Extracts were administered at different concentrations for 24 h prior to H_2_O_2_ 500 µM treatment for other 4 h. Cell viability data for (**A**) leaf (black) and (**B**) bark (blue) aqueous extracts of *A. occidentale* are shown. Data are presented as Mean ± SD of three independent experiments carried out in sextuplicate.

To in-depth evaluate the potential bioactivity of the most potent extracts of A. *occidentale* in depth, cytotoxicity assays were conducted in SH-SY5Y cells, a human neuroblastoma cell line commonly used in the context of oxidative stress and neurodegeneration [27,28,29] (Figure 5). The dose–response curve revealed that the LD_50_ values, which represent the concentration required to reduce cell viability to 50% compared to the control, were 275 µg/mL and 363 µg/mL for leaf and bark extracts, respectively.

**Figure 6:**
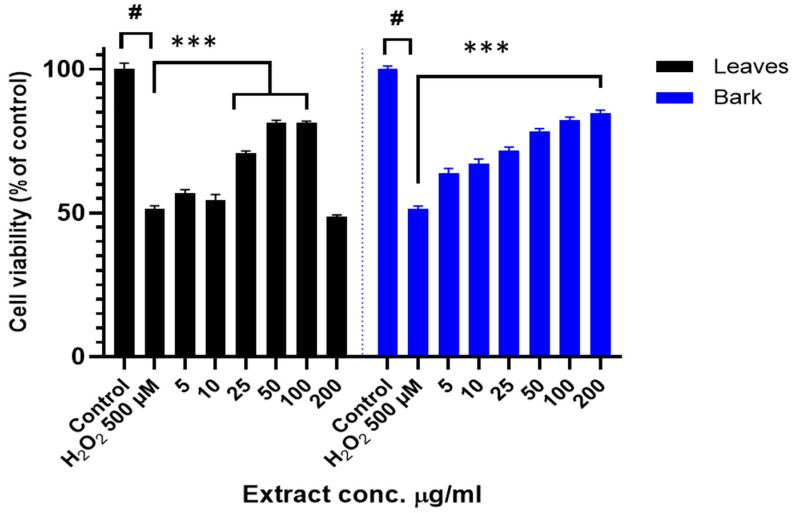
Neuroprotection effect of leaves and bark extracts against H_2_O_2_-induced cytotoxicity in SH-SY5Y cells. Cell viability was analyzed using MTT assay. Extracts were administered at different concentrations for 24 h prior to H_2_O_2_ 500 µM treatment for other 4 h. Cell viability data for leaf (black) and bark (blue) aqueous extracts of *A. occidentale* are shown. Data are presented as Mean ± SD of three independent experiments carried out in sextuplicate. # *p* < 0.001 vs. vehicle-treated control. *** *p* < 0.001 vs. only H_2_O_2_-treated cells.

Given the low toxicity of the extract, further experiments were carried out to evaluate the potential shielding capacity of the extracts in these human-origin neuronal cells. SH-SY5Y cells were treated with different concentrations of leaves or bark extracts (ranging from 5 to 200 µg/mL) for 24 h prior to H_2_O_2_ 500 µM treatment for another 4 h. As expected, H_2_O_2_ treatment significantly decreased the cell viability of SH-SY5Y (*p* < 0.05). Lower concentrations of the leaf extract could not effectively counteract the H_2_O_2_-derived cytotoxicity, while higher concentrations significantly restored cell viability (Figure 6). A dose-dependent experiment revealed similar behavior for the bark extract (Figure 6). Exposure to 500 µM H_2_O_2_ significantly reduced SH-SY5Y cell viability. When cells were pretreated with low concentrations of the aqueous bark extract, no significant protection against oxidative damage was observed. However, treatment with concentrations 10 µg/mL and above led to a statistically significant recovery of cell viability.

**Figure 7:**
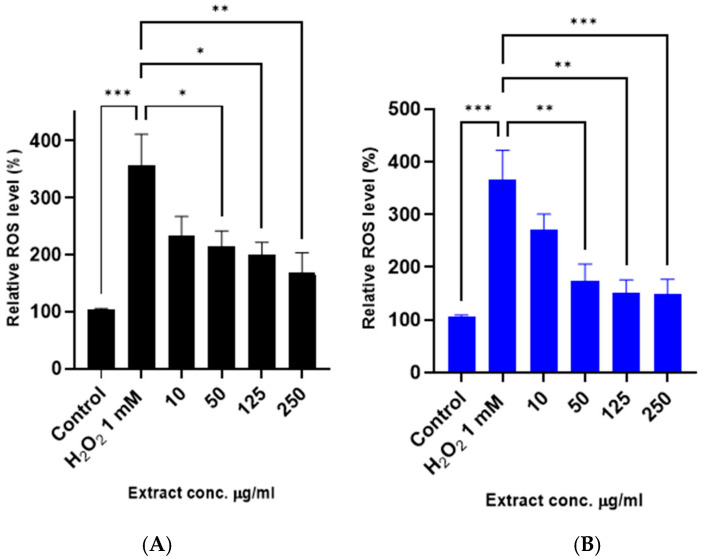
Reduction of intracellular reactive species production treated with leaf and bark extract against H_2_O_2_-induced cytotoxicity in SH-SY5Y cells. (**A**) Leaf (black) and (**B**) bark (blue) extracts inhibit H_2_O_2_ intracellular reactive species production. Cells were pretreated with the extracts before exposure to 1 mM H_2_O_2_ for 4 h, and intracellular reactive species levels were determined. Each bar represents the relative intracellular reactive species level expressed as percentage of control and significance of difference indicated with * (*p* < 0.05), ** (*p* < 0.01), *** (*p* < 0.001), when cells treated with extracts are compared to control and H_2_O_2_ vs. control cells.

As expected, under H_2_O_2_-derived oxidative stress, the level of reactive species increased dramatically. Both leaf and bark extracts effectively reduce intracellular reactive species levels in a dose-dependent manner (Figure 7). High levels of reactive species lead to a reduction in mitochondrial membrane potential, causing mitochondrial dysfunction and cell damage [3,27]. Thus, we evaluated the impact of leaf and bark extracts of *Anacardium occidentale* at the subcellular level using the fluorescent probe 5,5′,6,6′-tetrachloro-1,1′,3,3′-tetraethylbenzimidazolylcarbocyanine iodide (JC-1). JC-1 is commonly used to assess the polarization state of the mitochondrial membrane. In healthy mitochondria, JC-1 forms aggregates inside the organelle and displays red fluorescence. However, in depolarized mitochondria, the JC-1 dye fluoresces green and remains in the cytoplasm of the cells. Thus, the ratio of the red/green fluorescence allows us to infer mitochondrial function [28].

**Figure 8:**
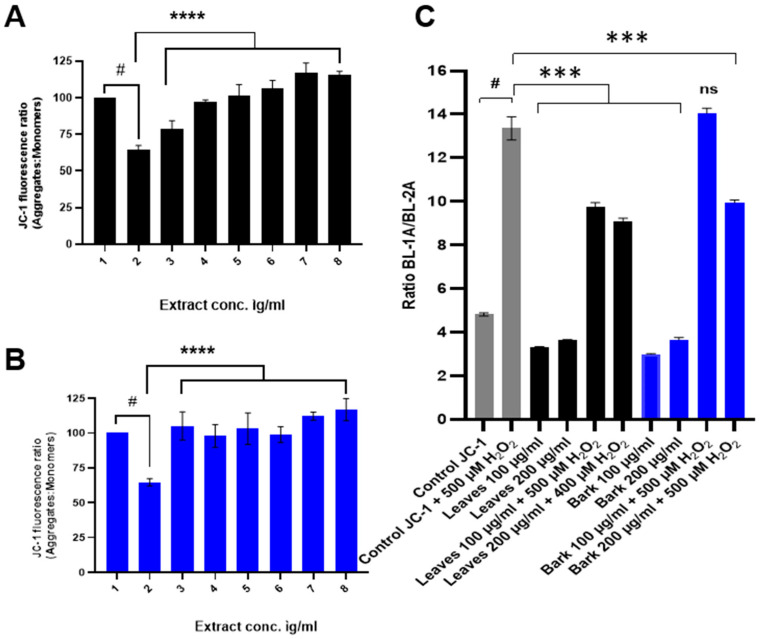
MMP loss prevention by leaves (**A**) and bark (**B**) extracts in SH-SH5Y under exposure to H_2_O_2_. Cells were treated with extracts for 24 h prior to exposure to 250 µM H_2_O_2_. MMP is expressed as the JC-1 fluorescence ratio in terms of red fluorescence to green fluorescence (Aggregates: Monomers). (**C**) Results from JC-1 flow cytometry experiments as the ratio of red to green fluorescence (BL-1a/BL-2A ratio). Cells were treated with extracts for 24 h prior to exposure to 500 µM H_2_O_2_. Data are presented as the Mean ± SEM of four independent experiments carried out in sextuplicate. # *p* < 0.001 vs. vehicle-treated control. *** *p* < 0.001, **** *p* < 0.0001 vs. cells treated only with H_2_O_2_. ns: not significant.

H_2_O_2_-treated SH-SY5Y cells reduced to 60% of the ratio of fluorescence when the dye was measured in 96-well plates (Figure 8A). Interestingly, pretreatment with any concentration of the leaf extract resulted in a significant recovery in the mitochondrial membrane potential. Also, pretreatment with bark extract at any tested concentration significantly alleviates the H_2_O_2_-mitochondrial derived damage. To confirm that the *A. occidentale* extract might provide mitochondrial protection, flow cytometry experiments using JC-1 were conducted. The FL1 channel captured the green fluorescence that represents damaged mitochondria, while the FL2 channel measured the JC-1 emission in red (healthy mitochondria). H_2_O_2_-treated cells showed an increase in the BL-1A/BL-2A ratio. Interestingly, the pretreatment with the plant extracts reduced the ratio (Figure 8C). In the experiment using leaf extract, both concentrations were successful in decreasing the BL-1A/BL-2A value. In contrast, only 200 µg/mL of bark extract was protective in terms of mitochondrial function in the flow cytometry analysis.

Altogether, these results indicate that leaf and bark extracts present low cytotoxicity and that SH-SY5Y cells exposed to these extracts are more resistant to H_2_O_2_-derived mitochondrial damage, counteracting oxidative stress.

### 3.4. Leaf and Bark Extracts (But Not Fruit and Nut Extracts) Trigger the Expression of NRF2-Target Cytoprotective Genes Against Oxidative Stress

While the direct ROS scavenger capacity of phytocompounds present in the extracts of the aerial parts of *A. occidentale* could be beneficial to combat cellular oxidative stress, the activation of cellular antioxidant response through the expression of cytoprotective genes could be a secondary protective mechanism.

**Figure 9:**
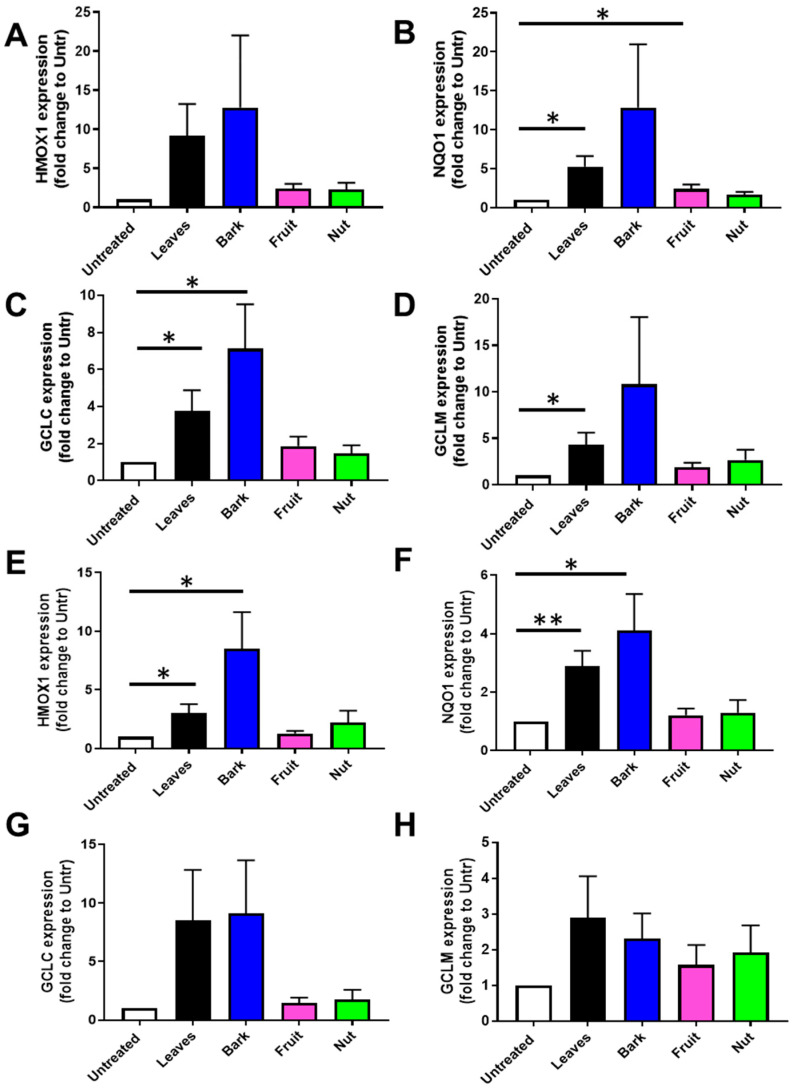
Leaves and bark aqueous extracts of *Anacardium occidentale* trigger the expression of NRF2-targets genes. Changes in NRF2-targets genes were analyzed in human lens epithelial cells (HLECs) and mouse embryonic fibroblasts (MEFs) treated for 24 h with 1 mg/mL of leaf (black), bark (blue), fruit (pink), and nut (green) aqueous extract. qPCR analysis was carried out against the indicated gene in (**A**–**D**) HLECs and (**E**–**H**) MEFs, n > 4; *, *p* < 0.05; **, *p* < 0.01, *t* test.

Given that natural compounds present in plant extracts have been shown to trigger the NRF2 signaling route, enhancing the function of antioxidant enzymes [30], we evaluated if extracts of different aerial parts of *A. occidentale* might induce the expression of well-described NRF2 target genes (Figure 9A–H and Figure S3). Both leaf and bark extracts impacted the expression of HMOX1, *NQO1, GCLC,* and *GCLM* in different cell lines, including MEFs, HLECs, and ARPE-19. On the contrary, neither fruit nor nut extracts significantly altered the expression of these cytoprotective genes.

Leaf and bark extracts induced the upregulation of *HMOX1* (Figure 9A,E). This transcriptional increment was statistically significant in MEFs cells (~3-fold change in leaves and 8-fold change compared to untreated cells), while high variability was found in HLECs exposed to these extracts (~9-fold change in leaves and 12-fold change compared to untreated cells). Fruit and nut extracts did not induce a significant increase in gene expression. Analogously, leaf extract promoted the upregulation of *NQO1* (~5-fold compared to the untreated group), and the bark extract resulted in the highest expression (~12-fold compared to untreated cells) in HLECs (Figure 9A). In MEFs, extracts from leaves and bark significantly induced the expression of the NQO1 gene (~3-fold change, *p* < 0.01, and ~4-fold change, *p* < 0.05, respectively) (Figure 9D).

In the case of genes related to glutathione metabolism, HLECs exposed to leaf and bark extracts displayed 4-fold and 7-fold increases in *GCLC*, respectively (Figure 9C). An approximately 8-fold increase in *GCLC* expression was detected in MEF cells treated with leaf and bark extracts, although the variability was higher in this cell type (Figure 9G). *GCLC* increased slightly in cells treated with fruit and cashew extracts, although this response was not statistically significant in any cell type. Although the upregulation of *GLCM* took place when both cell types were treated with leaf and bark extracts, only the aqueous leaf extract significantly stimulated *GLCM* in a statistically significant manner in HLECs (~4-fold change, *p* < 0.05). Neither fruit nor nut samples impacted the expression of this gene. Similar trends were also observed for all the analyzed genes in ARPE-19 cells (Figure S3).

Altogether, these results suggest that phytocompounds present in leaf and bark samples were able to transcriptionally modulate core genes involved in antioxidant defense.

## 4. Discussion

Aging is one of the most significant risk factors for the development of chronic neurodegenerative diseases, including Alzheimer’s disease, Parkinson’s disease, and amyotrophic lateral sclerosis, among others [2,3,4,5,6]. These disorders impose a considerable burden on the quality of life of affected individuals and represent a growing public health concern. At the cellular level, aging results from the cumulative impact of molecular damage over time, with oxidative stress playing a central role due to the excessive accumulation of reactive species. Therefore, understanding how oxidative stress contributes to the pathophysiology of aging and the onset of neurodegenerative diseases is essential. This highlights the urgent need to identify novel therapeutic strategies. In this context, the development of new antioxidant agents and identification of phytocompounds with antioxidant potential is crucial for advancing preventive and therapeutic approaches against neurodegeneration.

Although *A. occidentale* is widely used in herbal remedies by communities in tropical regions, its folkloric use is mainly based on perceived effectiveness against specific ailments, and there is a limited number of studies that provide research-based evidence to authenticate its pharmacological potential. Contemporary research provides fragmented knowledge on the different aerial parts of the plant, with numerous methods of extraction documented in the literature. Several studies have highlighted the nutraceutical potential of *A. occidentale*, particularly its edible parts and by-products. The cashew nut, a widely consumed food product, has demonstrated promising biological activities. In a murine model of colitis, oral administration of cashew nuts significantly ameliorated histological damage and reduced both inflammation and oxidative stress [31]. Similarly, in a dyslipidemic rat model, roasted cashew nuts reduced visceral fat accumulation, although an increase in glycemic levels and hepatic fat content was observed [32]. Interestingly, cashew nuts and their derivatives are also being evaluated in human clinical trials. For instance, the consumption of biscuits made with cashew nut flour led to a reduction in blood glucose levels and improved cholesterol markers in overweight and obese children [33]. Beyond the nut, the cashew apple and its by-products have also attracted attention due to their bioactive potential. These by-products have shown prebiotic effects by modulating the human gut microbiota as well as notable antimicrobial properties, suggesting their potential use as functional food additives [34,35]. Cashew apple juice, in particular, contains phytochemicals with antioxidant activity and wound-healing properties [36]. The bark of *A. occidentale* has also been proposed as a source of bioactive compounds with dermatological applications [37]. Traditional herbal preparations using the bark have demonstrated anti-inflammatory activity in carrageenan-induced rat paw edema models [38]. Among the various plant parts, the leaves appear to exhibit the greatest heterogeneity in terms of bioactive formulations reported in the literature, suggesting a broader therapeutic potential. For example, the administration of 100 mg/kg of ethanolic leaf extract in streptozotocin-induced diabetic rats produced antidiabetic effects comparable to those of pioglitazone, a standard antidiabetic drug [39]. Additionally, leaf extracts have shown antimalarial and antimicrobial activities [40,41]. Although numerous reports in the literature have explored the potential medicinal applications of different aerial parts of *Anacardium occidentale*, the diversity of extraction methods employed and wide range of experimental conditions make it challenging to determine which specific plant part holds the greatest therapeutic potential. Therefore, despite the promising therapeutic potential of *A. occidentale*, a comprehensive and systematic characterization of aerial part extracts has not been reported.

The literature on the chemical composition of *Anacardium occidentale* remains relatively limited, and interest in this species is growing, particularly regarding its nutritional profile and potential applications in the food industry [42]. Most studies on the chemical characterization of this plant have focused on the leaves, which appear to contain the highest concentration of bioactive compounds—a finding consistent with the results of the present study. For instance, Pham et al. identified 31 distinct compounds across various bioactive families, including terpenoids, phenolic compounds, and flavonoids, using different extraction methods applied to the leaves, including aqueous extracts [43]. Cashew apple juice has also been investigated for its potential in food formulation. In addition to its high carbohydrate content, it is notably rich in tannins and ascorbic acid [44]. Other plant parts, such as the bark, have also been chemically profiled. Ethyl acetate and ethanolic extracts of the bark have revealed the presence of bioactive compounds such as gallic acid, epigallocatechin gallate, and epicatechin [45].

**Figure 10:**
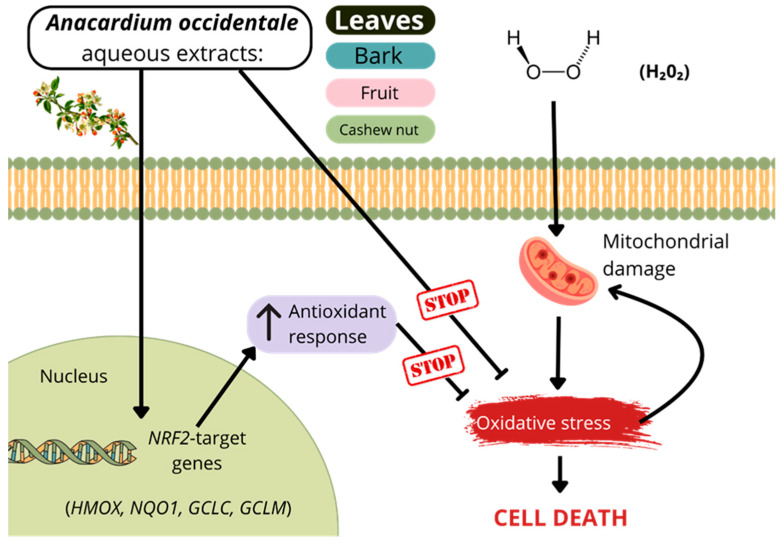
Working model. Leaves and bark (but not fruit and nut) aqueous extracts of *Anacardium occidentale* have high ROS scavenger capacity and trigger the expression of cytoprotective genes related to the NRF2 pathway.

Unlike previously published studies, our work provides a unique comprehensive analysis of the therapeutic potential of the different aerial parts of *Anacardium occidentale*, employing a multidisciplinary approach that integrates phytochemical profiling, antioxidant evaluation, and metabolomic analysis. This integrative strategy offers a broader and more robust understanding of the plant’s bioactive properties. The array of techniques applied in this study ranges from advanced metabolomics and electrochemical assays to biological tests designed to evaluate the capacity of the extracts to counteract oxidative stress. Our comparative analysis revealed that both leaf and bark aqueous extracts are not toxic and confer shielding capacity against oxidative stress (Figure 10). On the contrary, neither fruit nor nut aqueous extracts had a remarkable capacity to scavenge free radicals or trigger the expression of cytoprotective genes.

A battery of assays was used to provide phytochemical profiles and insights into the chemical compounds underlying the antioxidant properties of the different extracts of *A. occidentale.* Different metabolomic profiles were found, and the TPC assay revealed that the aqueous extract of *A. occidentale* leaves, and bark exhibited the highest polyphenol content. These results were further corroborated by metabolomic experiments, which detected the presence of trans-catechin, gallic, benzoic acid, or protocatechuic acid or pyrogallol. In alignment with our findings, other studies have identified similar polyphenols in mostly ethanolic extracts of nuts and leaves of *A. occidentale* or related species of the *Anacardiaceae* family [46,47,48]. In our study, cyclic voltammetry and spectrophotometric methods revealed a higher antioxidant capacity in both leaf and bark samples, thereby highlighting the potential therapeutic relevance of these aerial parts. Leaves are a well-recognized source of polyphenols, and the presence of bioactive compounds in leaves makes the consumption of leafy vegetables recommended due to their high nutritional benefits [49,50]. Bark extract displayed strong electrolytic activity, which may suggest a significant content of polyphenols, tannins, or flavonoids. As identified in our study, the presence of therapeutically active polyphenols in the bark of numerous plant species has been previously reported [51,52,53,54]. Thus, it is expected that there is a relative presence of polyphenols in leaf or bark extract, and our findings are consistent with other results found in the literature for different plant species. For example, leaf extracts of *Moringa oleifera* exhibit greater antioxidant power than stem extracts, with stem extracts in turn showing more antioxidant capacity than branch extracts, and *Barleria prionitis* L. leaves extracts have higher phenolic content and radical scavenger activity than flower and stem extracts [55,56].

On the contrary, metabolomic studies conducted with aqueous extracts of *A. occidentale* fruit revealed a less promising metabolomic profile in terms of potential antioxidant capacity and a minimal presence of total polyphenols in the sample. The FTIR assay suggests the presence of polysaccharides or organic acids, and, similarly, the spectrum obtained from the cyclic voltammetry assay indicates a high oxidation capacity of the sample, possibly due to the presence of a large quantity of oxidizable sugars. These compounds are consistent with those typically found in fruits, as they commonly accumulate high levels of sugars and organic acids such as citric acid, tartaric acid, and ascorbic acid. Also, these results may explain the presence of pectin, a polysaccharide with industrial and biotechnological interest, which has previously been identified in the fruit of *A. occidentale* [57]. The findings related to the cashew aqueous extract exhibited a pattern similar to that observed with the fruit. The FTIR and TPC analysis indicated the absence of polyphenolic compounds and a certain chemical diversity. Surprisingly, the scientific literature has not extensively documented the identification of pharmacologically relevant compounds in the cashew itself. Interestingly, anacardic acid—a molecule with promising therapeutic potential—is typically found in the cashew nutshell under normal conditions, rather than in the cashew kernel. Anacardic acid is thought to exert neuroprotective effects, modulate autophagic processes, and possess antibacterial properties [58,59,60,61]. Note that the limited activity in the aqueous extract of fruit or nuts could be attributed to the extraction method, as the presence of polyphenols in plant fruits is well-documented in the scientific literature [61]. It is possible that the polyphenols or other bioactive molecules present in the fruit and nuts are not fully water-soluble, or that the water temperature or pH may have hindered their extraction. Furthermore, the solvent used in maceration, or any extraction method, can be key for the obtention of molecules of interest. In this context, it may be of interest to explore alternative extraction methods, such as infusion, decoction, ultrasound-assisted extraction, or Soxhlet extraction in future experiments. Further experiments should be performed in the future using different organic solvents (or a mixture of these solvents) such as ethanol, hexane, ethyl acetate, or methanol.

In recent years, significant efforts have been devoted to identifying phytocompounds—particularly secondary metabolites—that may be useful in the context of nervous system-related or neurodegenerative diseases, where oxidative stress plays a critical role. In such scenarios, plant-derived compounds have gained special relevance due to their demonstrated strong antioxidant properties. In our study, further characterization revealed that the aqueous extracts from leaves and bark demonstrated a high degree of safety, exhibiting cytotoxicity only at very high concentrations, and were protective against oxidative stress and preserved proper mitochondrial function in vitro. In our study, leaf and bark extracts were neuroprotective in SH-SY5Y cells—a cell line commonly used as a model for the study of neurodegenerative diseases and disorders associated with the nervous system. Of note, there is increasing evidence that phytocompounds derived from the *Anacardiaceae* family might be neuroprotective. Methanolic bark extracts of *Anacardium microcarpum*, a species closely related to *A. occidentale*, provided neuroprotection in 6-hydroxydopamine-induced damage in chicken brain slices, and hydroalcoholic and methanolic extracts of the same plant demonstrated antioxidant and protective properties in vivo, showing protective effects against paraquat-induced stress in the *Drosophila melanogaster* model [62,63]. A hexanolic extract of the plant *Spondias mombin* (L.) (*Anacardiaceae*) has been shown to ameliorate oxidative status and provide neuroprotection at the behavioral level in zebrafish [64]. Vernicidin B, a flavonoid isolated from the ethanolic fraction of the plant *Toxicodendron vernicifluum* (*Anacardiaceae*), demonstrated neuroprotective properties by decreasing the production of ROS and reducing apoptosis induced by exposure to H_2_O_2_ [65]. Finally, the neuroprotective properties of leaf extracts of *A. occidentale* in vitro in HT22 and Neuro-2a cells were reported by improving neuroinflammation markers and various parameters related to neural function in rats [66,67].

Traditionally, the antioxidant capacity of a given plant extract has been directly linked, at least in part, to the polyphenol content of the sample. These bioactive molecules possess radical scavenging capacity and inherent antioxidant properties. However, a growing body of literature suggests that polyphenols can modulate different cellular processes, such as autophagy, fat and glucose metabolism, inflammation, and the response to oxidative stress [68]. The transcription factor NRF2 is a master regulator of genes related to the response to stress induced by free radicals. The development of therapeutic strategies targeting this transcription factor could be instrumental in addressing damage associated with oxidative stress, and as recently described, in neurodegenerative diseases [69]. The idea that plant extracts may activate the NRF2 route is a concept that is beginning to be explored in the field of phytotherapy. In our study, both leaf and bark extract enhanced NRF2-targets (*NQO1*, *HMOX1*, *GCLC,* and *GCLM*) in different cell types, suggesting that the impact of bioactive phytocompounds in the modulation of these cytoprotective genes is universal. In this regard, it is noteworthy that other plant extracts were reported to activate NRF2, including saponin extract of *Panax notoginseng*, olive leaf extract *Olea europaea*, or leaves’ ethanolic extract of *Rosmarinus officinalis* L. [70,71,72]. Nevertheless, although our findings suggest the importance of NRF2 as a mediator of the response derived from leaf and bark extracts of *A. occidentale*, we cannot discard a more complex scenario involving, for example, other transcription factors.

Taken together, our systematic characterization highlights extracts of *A. occidentale* arise as a potential therapeutic remedy against oxidative stress, within the context of neuroprotection. One of the upcoming challenges will be the identification, isolation, and validation of phytocompounds with high antioxidant power or the formulation of the extracts for therapeutic purposes. The development of these therapeutic strategies derived from *A. occidentale* might be a formulation to consider in pathological contexts with chronic oxidative stress, such as aging or aging-related diseases. Additionally, it is important to acknowledge one of the main limitations of this study: the biological activity of the extracts was assessed exclusively using immortalized cell lines and in vitro models. While these systems provide valuable preliminary insights, they do not fully replicate the complexity of living organisms. Therefore, future research should aim to evaluate the bioactive potential of the extracts in animal models, including assessments of in vivo toxicity and efficacy. These studies would lay the groundwork for subsequent clinical trials in humans, particularly in pathological contexts where oxidative stress plays a central role.

## Figures and Tables

**Table 1:** Primers used for quantitative real-time PCR assays.

Gene	Sequence (5′-3′)	Length (bp)	Tm (°C)	GC Content (%)	Species
*Nrf2 FW*	CAGAAGGAACAGGAGAAGGC	20	55.3	55	*Mus musculus*
*Nrf2 REV*	CTTGTTTGGGAATGTGGGC	19	54.6	52.6	*Mus musculus*
*Hmox FW*	TGCTCGAATGAACACTCTGG	20	54.9	50	*Mus musculus*
*Hmox1 REV*	TGGTCTTTGTGTTCCTCTGTC	21	54.7	47.6	*Mus musculus*
*Gclm FW*	ATGACCCGAAAGAACTGCTC	20	55	50	*Mus musculus*
*Gclm REV*	ATGATTCCCCTGCTCTTCAC	20	54.6	50	*Mus musculus*
*Gclc FW*	ACCATCACTTCATTCCCCAG	20	54.6	50	*Mus musculus*
*Gclc REV*	TTCTTGTTAGAGTACCGAAGCG	22	54.5	45.5	*Mus musculus*
*Nqo1 FW*	GGATTTGCCTACACATATGCTG	22	53.9	45.5	*Mus musculus*
*Nqo1 REV*	TGAATCGGCCAGAGAATGAC	20	54.8	50	*Mus musculus*
*Gapdh FW*	CCTGCTTCACCACCTTCTTGA	21	57.2	52.4	*Mus musculus*
*Gapdh REV*	TGTGTCCGTCGTGGATCTGA	20	58.1	55	*Mus musculus*
*NRF2 FW*	ATGACAATGAGGTTTCTTCGG	21	52.9	42.9	*Homo sapiens*
*NRF2 REV*	CAATGAAGACTGGGCTCTC	19	52.9	52.6	*Homo sapiens*
*HMOX1 FW*	AACTCCCTGGAGATGACTC	19	53.3	52.6	*Homo sapiens*
*HMOX1 REV*	CTCAAAGAGCTGGATGTTGAG	21	53.4	47.6	*Homo sapiens*
*GCLM FW*	GTTGACATGGCCTGTTCAG	19	53.9	52.6	*Homo sapiens*
*GCLM REV*	AACTCCATCTTCAATAGGAGGT	22	53.1	40.9	*Homo sapiens*
*GCLC FW*	AAGTGGATGTGGACACCAG	19	54.7	52.6	*Homo sapiens*
*GCLC REV*	CTGTCATTAGTTCTCCAGATGC	22	53.1	45.5	*Homo sapiens*
*NQO1 FW*	ACATCACAGGTAAACTGAAGG	21	52.3	42.9	*Homo sapiens*
*NQO1 REV*	TCAGATGGCCTTCTTTATAAGC	22	52.5	40.9	*Homo sapiens*
*GAPDH FW*	TCCTTCCTGGGCATGGAG	18	56.9	61.1	*Homo sapiens*
*GAPDH REV*	AGGAGGAGCAATGATCTTGATCTT	24	55.8	41.7	*Homo sapiens*

**Table 2:** Overview of the antioxidant parameters obtained by the ABTS, FRAP, and DPPH assays.

Sample	IC_50_ (µg/mL) (ABTS)	TEAC_50_ (ABTS)	Trolox Equivalents (FRAP)	IC_50_ (DPPH)
Trolox	5.8 ± 1.3	1	1	5.62
Leaves	12.3 ± 2.1	0.5	0.13	58.67
Bark	13.8 ± 4.1	0.4	0.12	79.52
Fruit	447.2 ± 15.0	0.01	0.03	>1000
Nut	>1000	n.d	0.01	>1000

## Data Availability

The original contributions presented in this study are included in the article/supplementary material. Further inquiries can be directed to the corresponding author(s).

## Supplementary Materials

The following supporting information can be downloaded at: https://www.mdpi.com/article/10.3390/antiox14080935/s1, Table S1. Metabolites identified in the *A. occidentale* leaf extract by GC × GC-TOFMS; Table S2. Metabolites identified in the *A. occidentale* bark extract by GC × GC-TOFMS; Table S3. Metabolites identified in the *A. occidentale* fruit extract by GC × GC-TOFMS; Table S4. Metabolites identified in the *A. occidentale* nut extract by GC × GC-TOFMS; Figure S1: GC × GC-TOFMS profiles of polyphenols identified in *A. occidentale* aqueous extracts; Figure S2: Separate graphs for the antioxidant capacity assays of aerial part of *A. occidentale*; Figure S3: Leaves and bark aqueous extracts of *A. occidentale* trigger the expression of NRF2-targets genes in retinal pigment epithelial cells.

## References

[1] World Health Organization (2019). Decade of Healthy Ageing: Proposal for a Decade of Action. WHO. https://www.who.int/docs/default-source/documents/decade-of-health-ageing/decade-ageing-proposal-en.pdf.

[2] Hajam Y.A., Rani R., Ganie S.Y., Sheikh T.A., Javaid D., Qadri S.S., Pramodh S., Alsulimani A., Alkhanani M.F., Harakeh S. (2022). Oxidative Stress in Human Pathology and Aging: Molecular Mechanisms and Perspectives. Cells.

[3] Yan M.H., Wang X., Zhu X. (2013). Mitochondrial defects and oxidative stress in Alzheimer disease and Parkinson disease. Free Radic. Biol. Med..

[4] Liguori I., Russo G., Curcio F., Bulli G., Aran L., Della-Morte D., Gargiulo G., Testa G., Cacciatore F., Bonaduce D. (2018). Oxidative stress, aging, and diseases. Clin. Interv. Aging.

[5] Yeung A.W.K., Tzvetkov N.T., Georgieva M.G., Ognyanov I.V., Kordos K., Jóźwik A., Kühl T., Perry G., Petralia M.C., Mazzon E. (2021). Reactive Oxygen Species and Their Impact in Neurodegenerative Diseases: Literature Landscape Analysis. Antioxid. Redox Signal..

[6] Böhm E.W., Buonfiglio F., Voigt A.M., Bachmann P., Safi T., Pfeiffer N., Gericke A. (2023). Oxidative stress in the eye and its role in the pathophysiology of ocular diseases. Redox Biol..

[7] Pizzino G., Irrera N., Cucinotta M., Pallio G., Mannino F., Arcoraci V., Squadrito F., Altavilla D., Bitto A. (2017). Oxidative Stress: Harms and Benefits for Human Health. Oxidative Med. Cell. Longev..

[8] Vomund S., Schäfer A., Parnham M.J., Brüne B., von Knethen A. (2017). Nrf2, the Master Regulator of Anti-Oxidative Responses. Int. J. Mol. Sci..

[9] Birben E., Sahiner U.M., Sackesen C., Erzurum S., Kalayci O. (2012). Oxidative stress and antioxidant defense. World Allergy Organ. J..

[10] Ngo V., Duennwald M.L. (2022). Nrf2 and Oxidative Stress: A General Overview of Mechanisms and Implications in Human Disease. Antioxidants.

[11] Schmidlin C.J., Dodson M.B., Madhavan L., Zhang D.D. (2019). Redox regulation by NRF2 in aging and disease. Free Radic. Biol. Med..

[12] Masyita A., Mustika Sari R., Dwi Astuti A., Yasir B., Rahma Rumata N., Emran T.B., Nainu F., Simal-Gandara J. (2022). Terpenes and terpenoids as main bioactive compounds of essential oils, their roles in human health and potential application as natural food preservatives. Food Chem. X.

[13] Heinrich M., Mah J., Amirkia V. (2021). Alkaloids Used as Medicines: Structural Phytochemistry Meets Biodiversity—An Update and Forward Look. Molecules.

[14] Rana A., Samtiya M., Dhewa T., Mishra V., Aluko R.E. (2022). Health benefits of polyphenols: A concise review. J. Food Biochem..

[15] Garnatje T., Peñuelas J., Vallès J. (2017). Ethnobotany, Phylogeny, and ‘Omics’ for Human Health and Food Security. Trends Plant Sci..

[16] González-Sarrías A., Núñez-Sánchez M.Á., Tomás-Barberán F.A., Espín J.C. (2017). Neuroprotective Effects of Bioavailable Polyphenol-Derived Metabolites against Oxidative Stress-Induced Cytotoxicity in Human Neuroblastoma SH-SY5Y Cells. J. Agric. Food Chem..

[17] Guerra R.N.M., Oliveira A.S., Farias J.R., Franco D.C.G., Santos P.G., Barbosa N.T., Muniz S.B., Abreu A.G., Nascimento F.R.F. (2025). Anacardiaceae Family: Effect of Isolated Compounds and Other Identified Phytochemicals Against Clinically Relevant Candida Species—A Short Review. Antibiotics.

[18] Méril-Mamert V., Ponce-Mora A., Sylvestre M., Lawrence G., Bejarano E., Cebrián-Torrejón G. (2022). Antidiabetic Potential of Plants from the Caribbean Basin. Plants.

[19] Ukwenya V.O., Alese M.O., Ogunlade B., Folorunso I.M., Omotuyi O.I. (2023). *Anacardium occidentale* leaves extract and riboceine mitigate hyperglycemia through anti-oxidative effects and modulation of some selected genes associated with diabetes. J. Diabetes Metab. Disord..

[20] Thesnor V., Molinié R., Giebelhaus R.T., de la Mata Espinosa A.P., Harynuk J.J., Bénimélis D., Vanhoye B., Dunyach-Rémy C., Sylvestre M., Cheremond Y. (2023). Antibacterial Activity and Untargeted Metabolomics Profiling of Acalypha arvensis Poepp. Molecules.

[21] Giebelhaus R.T., Nguyen G., Schmidt S.A., Wang S., Mesfin E.Y., Nam S.L., de la Mata A.P., Harynuk J.J. (2024). GC×GC-TOFMS Analysis of Fecal Metabolome Stabilized Using an At-Home Stool Collection Device. Appl. Biosci..

[22] Sumner L.W., Amberg A., Barrett D., Beale M.H., Beger R., Daykin C.A., Fan T.W., Fiehn O., Goodacre R., Griffin J.L. (2007). Proposed minimum reporting standards for chemical analysis Chemical Analysis Working Group (CAWG) Metabolomics Standards Initiative (MSI). Metabolomics.

[23] Nam S.L., Giebelhaus R.T., Tarazona Carrillo K.S., de la Mata A.P., Harynuk J.J. (2024). Evaluation of normalization strategies for GC-based metabolomics. Metabolomics.

[24] Brinvillier D., Barrast M., Couderc-Murillo P., Bono-Yagüe J., Rousteau A., Gómez Escribano A.P., Palmeira-Mello M.V., Doménech-Carbó A., Passe-Coutrin N., Sylvestre M. (2022). Spectroscopic, Electrochemical, and Biological Assays of Copper-Binding Molecules for Screening of Different Drugs and Plant Extracts against Neurodegenerative Disorders. ACS Omega.

[25] Matignon L., Lo M.M., Monpierre M., Correia M.V., Valencia D.P., Palmeira-Mello M.V., Sylvestre M.-N., Pruneau L., Sylvestre M., Domenech A. (2023). Phytochemical and Biological Study of Trophic Interaction between *Pseudosphinx tetrio* L. Larvae and *Allamanda cathartica* L.. Plants.

[26] Corpas F.J., Rodríguez-Ruiz M., Campos M.J., Taboada J., Palma J.M. (2024). Electrochemical Detection of Total Antioxidant Capacity (TAC) in Plant Tissues from Different Origins. Methods Mol. Biol..

[27] Nieto C.I., Cornago M.P., Cabildo M.P., Sanz D., Claramunt R.M., Torralba M.C., Torres M.R., Martínez Casanova D., Sánchez-Alegre Y.R., Escudero E. (2018). Evaluation of the Antioxidant and Neuroprotectant Activities of New Asymmetrical 1,3-Diketones. Molecules.

[28] Angelé-Martínez C., Nguyen K.V., Ameer F.S., Anker J.N., Brumaghim J.L. (2017). Reactive oxygen species generation by copper(II) oxide nanoparticles determined by DNA damage assays and EPR spectroscopy. Nanotoxicology.

[29] Pang Q.Q., Kim J.H., Kim H.Y., Kim J.-H., Cho E.J. (2023). Protective Effects and Mechanisms of Pectolinarin against H_2_O_2_-Induced Oxidative Stress in SH-SY5Y Neuronal Cells. Molecules.

[30] Wang T., Liu M., Li X., Zhang S., Gu H., Wei X., Wang X., Xu Z., Shen T. (2024). Naturally-derived modulators of the Nrf2 pathway and their roles in the intervention of diseases. Free Radic. Biol. Med..

[31] Siracusa R., Fusco R., Peritore A.F., Cordaro M., D’Amico R., Genovese T., Gugliandolo E., Crupi R., Smeriglio A., Mandalari G. (2020). The Antioxidant and Anti-Inflammatory Properties of *Anacardium occidentale* L. Cashew Nuts in a Mouse Model of Colitis. Nutrients.

[32] Dias C.C.Q., Madruga M.S., Pintado M.M.E., Almeida G.H.O., Alves A.P.V., Dantas F.A., Bezerra J.K.G., de Melo M., Viera V.B., Soares J.K.B. (2019). Cashew nuts (*Anacardium occidentale* L.) decrease visceral fat, yet augment glucose in dyslipidemic rats. PLoS ONE.

[33] Brito L.B., Leal B.O., Silva J.R.D., Barbosa K.M.P., Silva V.T.D., Costa A.S., Landim Y.P., Pascoal L.M., Neto M.S., Pereira A.L.F. (2025). Effect of the consumption of cashew nut (*Anacardium occidentale* L.) flour-based biscuits in overweight children: A pilot randomized clinical trial. Efecto del consumo de galletas a base de harina de anacardo (*Anacardium occidentale* L.) en niños con sobrepeso: Un ensayo clínico piloto aleatorizado. Nutr. Hosp..

[34] Menezes F., da Cruz Almeida É.T., da Silva Vieira A.R., de Souza Aquino J., Dos Santos Lima M., Magnani M., de Souza E.L. (2021). Impact of Cashew (*Anacardium occidentale* L.) by-Product on Composition and Metabolic Activity of Human Colonic Microbiota In Vitro Indicates Prebiotic Properties. Curr. Microbiol..

[35] Sousa J.M.S., de Abreu F.A.P., Ruiz A.L.T.G., da Silva G.G., Machado S.L., Garcia C.P.G., Filho F.O., Wurlitzer N.J., de Figueiredo E.A.T., Magalhães F.E.A. (2021). Cashew apple (*Anacardium occidentale* L.) extract from a by-product of juice processing: Assessment of its toxicity, antiproliferative and antimicrobial activities. J. Food Sci. Technol..

[36] da Silveira Vasconcelos M., Gomes-Rochette N.F., de Oliveira M.L., Nunes-Pinheiro D.C., Tomé A.R., Maia de Sousa F.Y., Pinheiro F.G., Moura C.F., Miranda M.R., Mota E.F. (2015). Anti-inflammatory and wound healing potential of cashew apple juice (*Anacardium occidentale* L.) in mice. Exp. Biol. Med..

[37] Borges J. (2021). Cashew tree (*Anacardium occidentale*): Possible applications in dermatology. Clin. Dermatol..

[38] Encarnação S., Lima K., Malú Q., Caldeira G.I., Duarte M.P., Rocha J., Lima B.S., Silva O. (2024). An Integrated Approach to the Anti-Inflammatory, Antioxidant, and Genotoxic Potential of Portuguese Traditional Preparations from the Bark of *Anacardium occidentale* L.. Plants.

[39] Jaiswal Y.S., Tatke P.A., Gabhe S.Y., Vaidya A.B. (2017). Antidiabetic activity of extracts of *Anacardium occidentale* Linn. leaves on n-streptozotocin diabetic rats. J. Tradit. Complement. Med..

[40] Kaushik M., Hoti S.L., Saxena J.K., Hingamire T., Shanmugam D., Joshi R.K., Metgud S.C., Ungar B., Singh I., Hegde H.V. (2023). Antimalarial Activity of *Anacardium occidentale* Leaf Extracts Against Plasmodium falciparum Transketolase (PfTK). Acta Parasitol..

[41] Sunderam V., Thiyagarajan D., Lawrence A.V., Mohammed S.S.S., Selvaraj A. (2019). In-vitro antimicrobial and anticancer properties of green synthesized gold nanoparticles using *Anacardium occidentale* leaves extract. Saudi J. Biol. Sci..

[42] Salehi B., Gültekin-Özgüven M., Kırkın C., Özçelik B., Morais-Braga M.F.B., Carneiro J.N.P., Bezerra C.F., Silva T.G.D., Coutinho H.D.M., Amina B. (2019). Anacardium Plants: Chemical, Nutritional Composition and Biotechnological Applications. Biomolecules.

[43] Pham D.C., Truong D.H., Tran Q.H., Ho Q.T., Nguyen T.A.D., Nguyen T.N.H., Nguyen T.V., Nguyen T.T.V., Cao T.S., Barrow C.J. (2023). Fractionation, identification of chemical constituents, and biological properties of cashew (*Anacardium occidentale* L.) leaf extracts. Food Sci. Nutr..

[44] Cruz Reina L.J., Durán-Aranguren D.D., Forero-Rojas L.F., Tarapuez-Viveros L.F., Durán-Sequeda D., Carazzone C., Sierra R. (2022). Chemical composition and bioactive compounds of cashew (*Anacardium occidentale*) apple juice and bagasse from Colombian varieties. Heliyon.

[45] de Almeida M.M., Rosa-Rezende M.A., Azevedo M.B., de Oliveira E.A.M., de Castro S.B.R., Alves C.C.S., Cabrera G.M., Siless G., Lang K.L., Ferreira G.F. (2025). Unveiling the Chemical Composition and the Antifungal Mechanisms of a Phenolic-rich Fraction of *Anacardium occidentale* L. Bark. Chem. Biodivers..

[46] Sruthi P., Roopavathi C., Madhava Naidu M. (2023). Profiling of phenolics in cashew nut (*Anacardium occidentale* L.) testa and evaluation of their antioxidant and antimicrobial properties. Food Biosci..

[47] Désiré G.N.S., Simplice F.H., Guillaume C.W., Kamal F.Z., Parfait B., Hermann T.D.S., Hervé N.A.H., Eglantine K.W., Linda D.K.J., Roland R.N. (2023). Cashew (*Anacardium occidentale*) Extract: Possible Effects on Hypothalamic–Pituitary–Adrenal (HPA) Axis in Modulating Chronic Stress. Brain Sci..

[48] Lima Júnior J.P.d., Franco R.R., Saraiva A.L., Moraes I.B., Espindola F.S. (2021). *Anacardium humile* St. Hil as a novel source of antioxidant, antiglycation and α-amylase inhibitors molecules with potential for management of oxidative stress and diabetes. J. Ethnopharmacol..

[49] Amat-ur-Rasool H., Symes F., Tooth D., Schaffert L.-N., Elmorsy E., Ahmed M., Hasnain S., Carter W.G. (2020). Potential Nutraceutical Properties of Leaves from Several Commonly Cultivated Plants. Biomolecules.

[50] Masiala A., Vingadassalon A., Aurore G. (2024). Polyphenols in edible plant leaves: An overview of their occurrence and health properties. Food Funct..

[51] Yazaki Y. (2015). Utilization of flavonoid compounds from bark and wood: A review. Nat. Prod. Commun..

[52] Faggian M., Bernabè G., Ferrari S., Francescato S., Baratto G., Castagliuolo I., Dall’Acqua S., Peron G. (2021). Polyphenol-Rich Larix decidua Bark Extract with Antimicrobial Activity against Respiratory-Tract Pathogens: A Novel Bioactive Ingredient with Potential Pharmaceutical and Nutraceutical Applications. Antibiotics.

[53] Wafa D., Nicolas B. (2020). Pine Bark Phenolic Extracts, Current Uses, and Potential Food Applications: A Review. Curr. Pharm. Des..

[54] Her Y., Lee T.K., Sim H., Lee J.C., Kim D.W., Choi S.Y., Hong J.K., Lee J.W., Kim J.D., Won M.H. (2022). *Pinus thunbergii* bark extract rich in flavonoids promotes hair growth in dorsal skin by regulating inflammatory cytokines and increasing growth factors in mice. Mol. Med. Rep..

[55] Shih M.-C., Chang C.-M., Kang S.-M., Tsai M.-L. (2011). Effect of Different Parts (Leaf, Stem and Stalk) and Seasons (Summer and Winter) on the Chemical Compositions and Antioxidant Activity of *Moringa oleifera*. Int. J. Mol. Sci..

[56] Sunil K.J., Mukesh K.D., Sanjeeb D., Arti Raj V., Ch V.R. (2010). A comparative study on total phenolic content, reducing power and free radical scavenging activity of aerial parts of *Barleria prionitis*. Int. J. Phytomed..

[57] Tamiello-Rosa C.S., Cantu-Jungles T.M., Iacomini M., Cordeiro L.M.C. (2019). Pectins from cashew apple fruit (*Anacardium occidentale*): Extraction and chemical characterization. Carbohydr. Res..

[58] da Silva W.M.B., Pinheiro S.O., Alves D.R., de Menezes J., Magalhães F.E.A., Silva F.C.O., Marinho M.M., Marinho E.S., de Morais S.M. (2021). Anacardic Acid Complexes as Possible Agents Against Alzheimer’s Disease Through Their Antioxidant, In vitro, and In silico Anticholinesterase and Ansiolic Actions. Neurotox. Res..

[59] Seong Y.A., Shin P.G., Yoon J.S., Yadunandam A.K., Kim G.D. (2014). Induction of the endoplasmic reticulum stress and autophagy in human lung carcinoma A549 cells by anacardic acid. Cell Biochem. Biophys..

[60] Hollands A., Corriden R., Gysler G., Dahesh S., Olson J., Raza Ali S., Kunkel M.T., Lin A.E., Forli S., Newton A.C. (2016). Natural Product Anacardic Acid from Cashew Nut Shells Stimulates Neutrophil Extracellular Trap Production and Bactericidal Activity. J. Biol. Chem..

[61] Williamson G. (2017). The role of polyphenols in modern nutrition. Nutr. Bull..

[62] Martins I.K., de Carvalho N.R., Macedo G.E., Rodrigues N.R., Ziech C.C., Vinadé L., Filho V.M.B., Menezes I.A., Franco J., Posser T. (2018). *Anacardium microcarpum* Promotes Neuroprotection Dependently of AKT and ERK Phosphorylation but Does Not Prevent Mitochondrial Damage by 6-OHDA. Oxidative Med. Cell. Longev..

[63] Müller K.R., Martins I.K., Rodrigues N.R., da Cruz L.C., Barbosa Filho V.M., Macedo G.E., da Silva G.F., Kamdem J.P., de Menezes I.R.A., Franco J.L. (2017). *Anacardium microcarpum* extract and fractions protect against paraquat-induced toxicity in Drosophila melanogaster. EXCLI J..

[64] Santo G.D., de Veras B.O., Rico E., Magro J.D., Agostini J.F., Vieira L.D., Calisto J.F.F., Mocelin R., de Sá Fonseca V., Wanderley A.G. (2021). Hexane extract from *SpoSndias mombin* L. (Anacardiaceae) prevents behavioral and oxidative status changes on model of Parkinson’s disease in zebrafish. Comp. Biochem. Physiology. Toxicol. Pharmacol. CBP.

[65] Zhong T., Li M., Wu H., Wang D., Liu J., Xu Y., Fan Y. (2022). Novel Flavan-3,4-diol vernicidin B from Toxicodendron Vernicifluum (Anacardiaceae) as potent antioxidant via IL-6/Nrf2 cross-talks pathways. Phytomedicine.

[66] Oyagbemi A.A., Kolawole A.A., Elizabeth A.O., Oluwaseun A.K., Racheal F.O., Olanrewaju E.O., Olabisi A.T., Seun O.B., Olubunmi F.O., Omolola O.I. (2023). Leaf extract of *Anacardium occidentale* ameliorates biomarkers of neuroinflammation, memory loss, and neurobehavioral deficit in N(ω)-nitro-L-arginine methyl ester (L-NAME) treated rats. Biomarkers.

[67] Duangjan C., Rangsinth P., Zhang S., Wink M., Tencomnao T. (2021). *Anacardium occidentale* L. Leaf Extracts Protect Against Glutamate/H_2_O_2_-Induced Oxidative Toxicity and Induce Neurite Outgrowth: The Involvement of SIRT1/Nrf2 Signaling Pathway and Teneurin 4 Transmembrane Protein. Front. Pharmacol..

[68] Ponce-Mora A., Salazar N.A., Domenech-Bendaña A., Locascio A., Bejarano E., Gimeno-Mallench L. (2025). Interplay Between Polyphenols and Autophagy: Insights From an Aging Perspective. Front. Biosci. (Landmark Ed.).

[69] Mayer C., Riera-Ponsati L., Kauppinen S., Klitgaard H., Erler J.T., Hansen S.N. (2024). Targeting the NRF2 pathway for disease modification in neurodegenerative diseases: Mechanisms and therapeutic implications. Front. Pharmacol..

[70] Zhao Y., Zheng G., Yang S., Liu S., Wu Y., Miao Y., Liang Z., Hua Y., Zhang J., Shi J. (2024). The plant extract PNS mitigates atherosclerosis via promoting Nrf2-mediated inhibition of ferroptosis through reducing USP2-mediated Keap1 deubiquitination. Br. J. Pharmacol..

[71] HAS A.L., Alotaibi M.F., Bin-Jumah M., Elgebaly H., Mahmoud A.M. (2019). Olea europaea leaf extract up-regulates Nrf2/ARE/HO-1 signaling and attenuates cyclophosphamide-induced oxidative stress, inflammation and apoptosis in rat kidney. Biomed. Pharmacother..

[72] Wang H., Cheng J., Yang S., Cui S.W., Wang M., Hao W. (2020). Rosemary extract reverses oxidative stress through activation of Nrf2 signaling pathway in hamsters fed on high fat diet and HepG2 cells. J. Funct. Foods.

