# Supplementary figures and images for: Systematic Characterization of Antioxidant Shielding Capacity Against Oxidative Stress of Aerial Part Extracts of Anacardium occidentale
Source: Antioxidants (Basel). 2025 Jul 30;14(8):935. doi: 10.3390/antiox14080935 (PMC12382801; doi:10.3390/antiox14080935)

**A**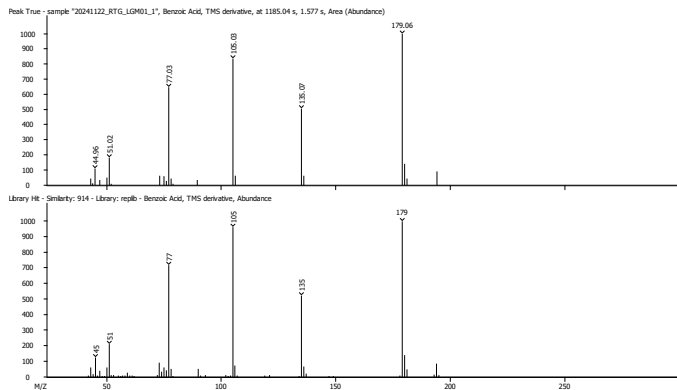**B**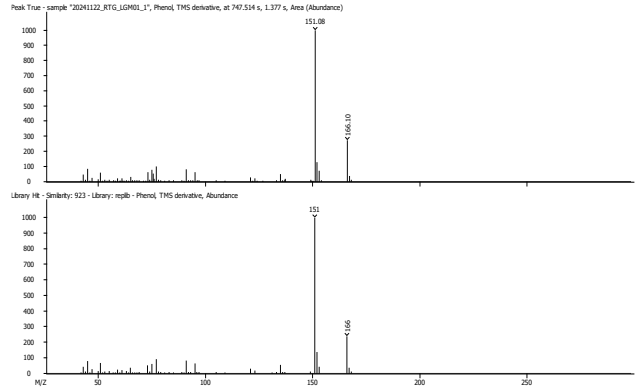**C**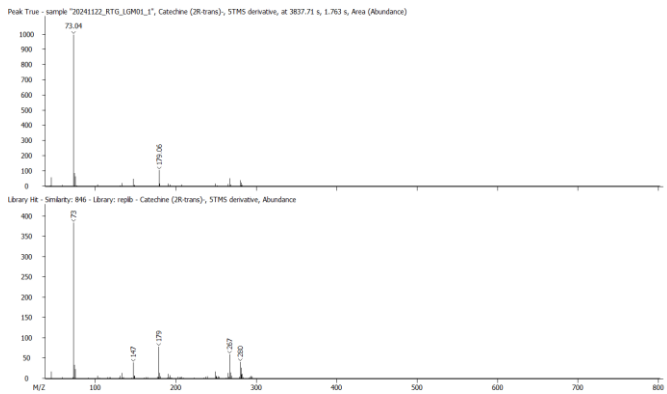**D**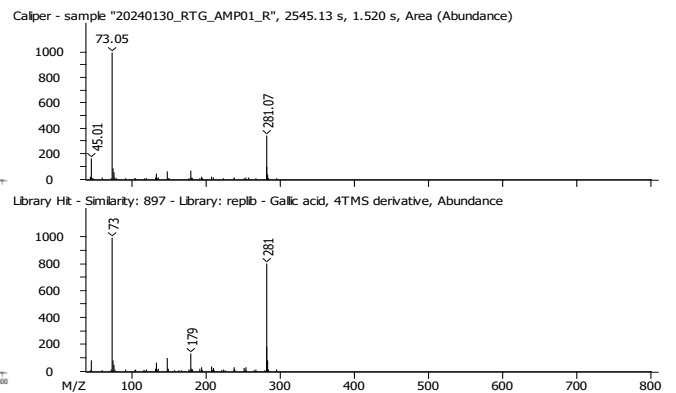**E**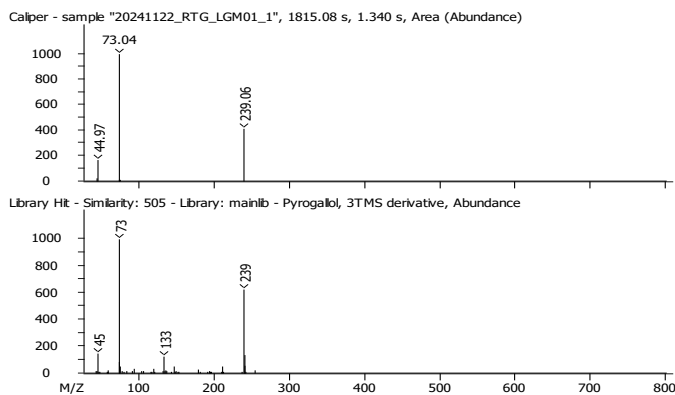**F**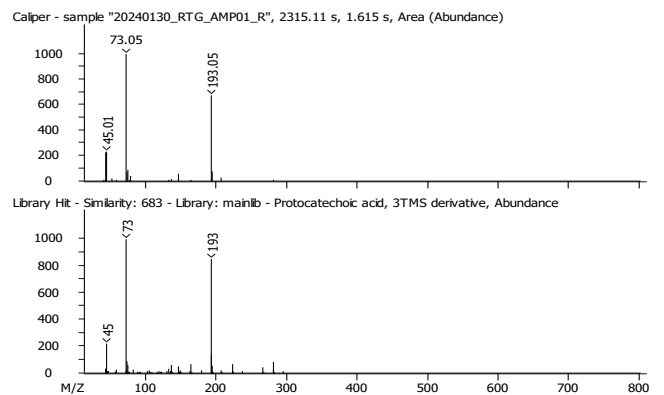

Supplement: Supplementary file 1 [file antioxidants-14-00935-s001.zip › Supp Figure S1.pdf]

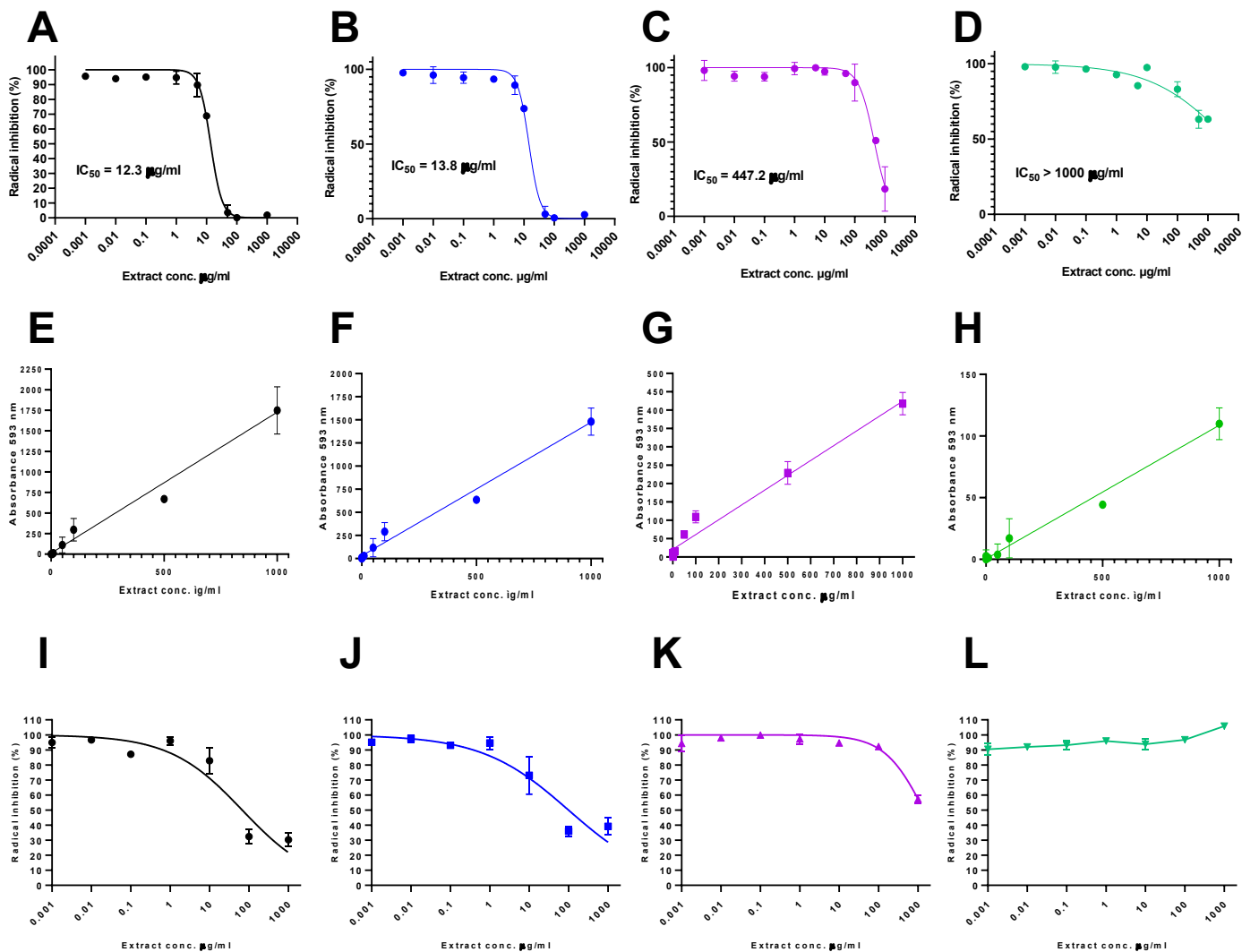

Supplement: Supplementary file 1 [file antioxidants-14-00935-s001.zip › Supp Figure S2.pdf]

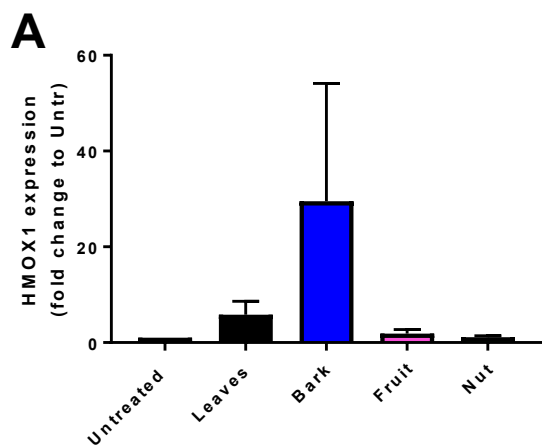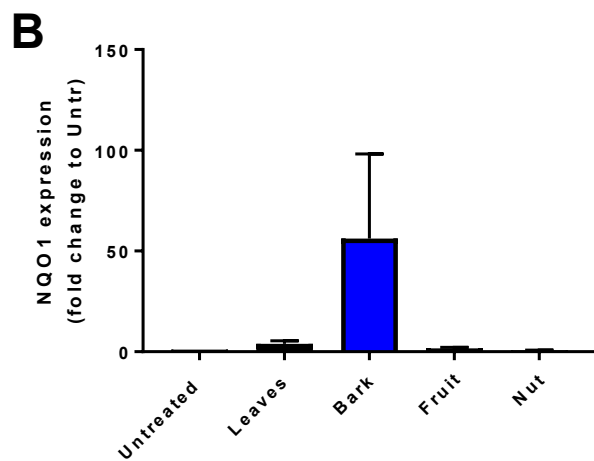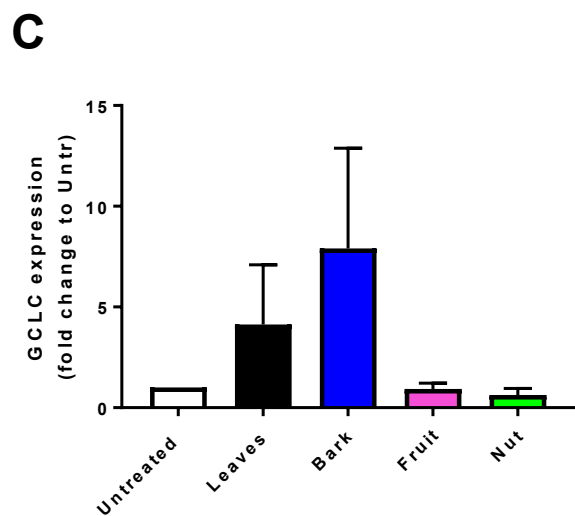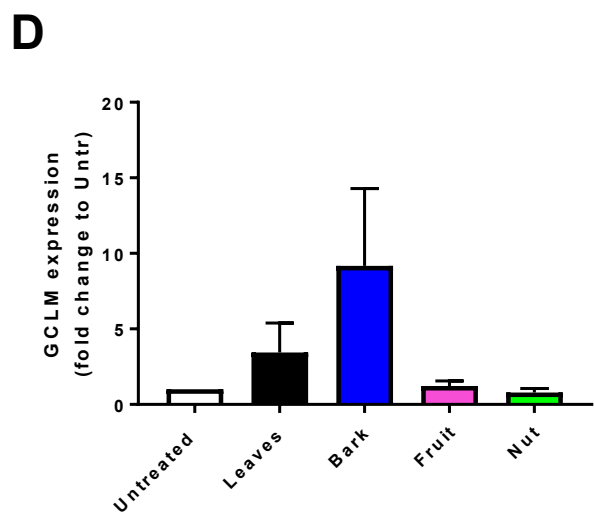

Supplement: Supplementary file 1 [file antioxidants-14-00935-s001.zip › Suppl Figure S3.pdf]
